# Wingless ligand 5a is a critical regulator of placental growth and survival

**DOI:** 10.1038/srep28127

**Published:** 2016-06-17

**Authors:** Gudrun Meinhardt, Leila Saleh, Gerlinde R. Otti, Sandra Haider, Philipp Velicky, Christian Fiala, Jürgen Pollheimer, Martin Knöfler

**Affiliations:** 1 Department of Obstetrics and Gynecology, Reproductive Biology Unit; Medical University of Vienna, Vienna, Austria; 2 Gynmed Clinic, Vienna, Austria

## Abstract

The maternal uterine environment is likely critical for human placental morphogenesis and development of its different trophoblast subtypes. However, factors controlling growth and differentiation of these cells during early gestation remain poorly elucidated. Herein, we provide evidence that the ligand Wnt5a could be a critical regulator of trophoblast proliferation and survival. Immunofluorescence of tissues and western blot analyses of primary cultures revealed abundant Wnt5a expression and secretion from first trimester decidual and villous stromal cells. The ligand was also detectable in decidual glands, macrophages and NK cells. Wnt5a increased proliferation of villous cytotrophoblasts and cell column trophoblasts, outgrowth on collagen I as well as cyclin A and D1 expression in floating explant cultures, but suppressed camptothecin-induced apoptosis. Similarly, Wnt5a stimulated BrdU incorporation and decreased caspase-cleaved cytokeratin 18 neo-epitope expression in primary cytotrophoblasts. Moreover, Wnt5a promoted activation of the MAPK pathway in the different trophoblast models. Chemical inhibition of p42/44 MAPK abolished cyclin D1 expression and Wnt5a-stimulated proliferation. Compared to controls, MAPK phosphorylation and proliferation of cytotrophoblasts declined upon supplementation of supernatants from Wnt5a gene-silenced decidual or villous stromal cells. In summary, non-canonical Wnt5a signalling could play a role in early human trophoblast development by promoting cell proliferation and survival.

Rapid development of placental structures during the first weeks of gestation is critical for embryonic survival and maintenance of pregnancy. Whereas cytotrophoblast (CTB) progenitors in floating placental villi differentiate into the multinucleated syncytium, proliferative CTBs of anchoring villi give rise to extravillous trophoblasts (EVT) invading the maternal uterus. Besides a strong intrinsic molecular program generating the diverse specialized trophoblast subtypes, endocrine secretions from uterine cells are likely important for trophoblast growth and branching morphogenesis of the human placenta during the first weeks of gestation^1^,^2^. Shortly after implantation cytotrophoblasts (CTBs) of primary villi contact the decidua and expand laterally thereby forming the cytotrophoblastic shell protecting the embryo from early insults of the maternal environment such as oxidative stress^3^. Formations of channels through the shell connecting decidual glands with the developing intervillous space suggested that glandular secretions could be necessary for histiotrophic nutrition of the fetus as well as for early stages of trophoblast development^4^,^5^. Indeed, glandular epithelial cells of the decidua are rich in carbohydrates and lipids but also produce a variety of growth factors, such as epidermal growth factor, stimulating trophoblast proliferation *in vitro*^6^,^7^. At later stages of pregnancy, formation of anchoring villi, attaching to the decidua, and invasion of CTBs into uterine tissue and vessels takes place. The latter contributes to spiral artery remodeling and establishment of the haemotrophic nutrition^8^, and is likely controlled by diverse decidual cell types. Indeed, numerous autocrine as well as paracrine factors present at the fetal-maternal interface could regulate trophoblast proliferation, differentiation and migration^9^. Defects in placentation, provoking abnormal uterine vessel remodeling, trophoblast invasion and survival, were associated with different pregnancy disorders, such as miscarriage, preterm labour and preeclampsia^10^,^11^,^12^. Besides failures in the intrinsic EVT differentiation program, deranged uterine secretions could be an underlying cause^4^,^13^. However, the network of soluble decidual factors controlling placentation and its role in the pathogenesis of gestational diseases remain poorly understood.

Recently, the developmental Wingless (Wnt) pathway, which regulates stem cell maintenance, cell fate decisions and differentiation during embryogenesis and in adult tissues^14^, has also been implicated in endometrial function, implantation and placental development^15^. Activation of canonical Wnt signalling requires binding of secreted Wnt ligands to hetero-dimeric receptors consisting of frizzled (Fzd) family members and low-density lipoprotein receptor related protein 5 or 6 (LRP-5/6)^16^. As a consequence, cytosolic β-catenin is stabilized by dissolving its destruction complex and recruited to the nucleus^14^. Active β-catenin then binds to nuclear factors of the lymphoid enhancer binding factor-1 (LEF-1)/T-cell factor (TCF) family thereby activating transcription of genes which promote cell cycle progression, differentiation and invasion^16^. Whereas studies in mice indicated a role of the Wnt pathway in early trophectoderm specification, chorio-allantoic fusion, placental branching morphogenesis and labyrinth development^17^,^18^, canonical signalling in humans was shown to enhance syncytialisation of choriocarcinoma cells as well as EVT differentiation and migration^19^,^20^,^21^. Noteworthy, first trimester CTBs produce numerous Wnt molecules and Fzd receptors and the soluble Wnt inhibitor Dickkopf-1 (Dkk1) reduced basal trophoblast motility indicating autocrine effects^20^,^22^. On the other hand, human endometrial cells express various Wnt ligands^23^,^24^ suggesting that paracrine activation of the pathway could also regulate trophoblast development and function. However, expression patterns of Wnt ligands in the diverse decidual cell types of early pregnancy and their individual roles in the cross-talk with placental trophoblast remain largely elusive.

Among the different ligands Wnt5a might have a pivotal function in reproduction since it is required for the development of uterine glands and the posterior region of the female reproductive tract and also regulates blastocyst implantation^25^,^26^. Although Wnt5a can activate LEF-1/TCF/β-catenin-mediated transcription^27^, it antagonizes the canonical Wnt pathway in most cellular systems and provokes non-canonical signalling through a variety of different cascades^28^. Besides elevating intracellular Ca^2+^ concentrations^29^, Wnt5a was shown to activate RhoA/Rho kinase, Rac/c-Jun terminal kinase, phosphatidylinositide 3-kinase (PI3K)/AKT and mitogen-activated protein kinase (MAPK)^30^,^31^,^32^.

Recent evidence from our laboratory suggested that Wnt5a also suppresses canonical Wnt signalling in trophoblasts^22^. Since the latter pathway was associated with EVT functions^19^,^20^, we speculated that non-canonical signalling through Wnt5a could control other aspects of placental morphogenesis such as growth of villous CTBs and cell columns. Therefore, we herein investigated decidual and placental expression patterns of Wnt5a by immunofluorescence and western blotting and studied its function using different primary trophoblast models. The data suggest that Wnt5a, secreted by villous and decidual stromal cells, promotes trophoblast proliferation and survival involving activation of the MAPK pathway.

## Results

### Wnt5a is widely expressed in first trimester placental and decidual cells

**Figure 1:**
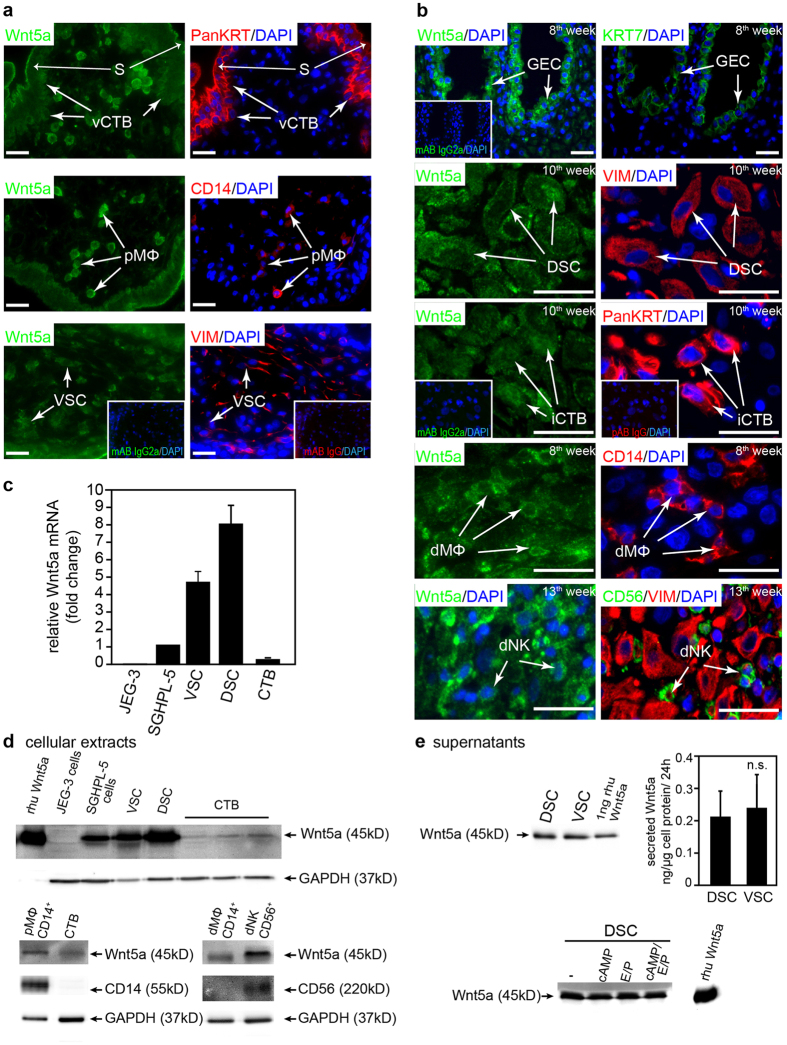
Expression of Wnt5a in tissue sections and primary cultures of first trimester placenta and decidua. (**a**) Immunofluorescence of 11^th^ week placenta. Representative slides taken from three different first trimester tissues are shown. Antibodies recognizing Wnt5a, pan-keratin (PanKRT, villous trophoblasts), vimentin (VIM, stromal cells), cytokeratin 7 (KRT7, glandular epithelial cells), CD14 (macrophages) and CD56 (decidual NK cells) were utilized. In negative controls (insert pictures) primary antibodies were replaced by rabbit polyclonal IgG (pAB IgG), or mouse or rabbit monoclonal isotype controls (mAB IgG). Scale bars represent 50 μm. vCTB, villous cytotrophoblast; pMΦ, placental macrophage; VSC, villous stromal cell; S, syncytium; (**b**) Immunofluorescence in first trimester decidua (8–13^th^ week). Representative pictures of four different tissues are shown. CD56 immunofluorescence (13^th^ week) was performed on a serial section. GEC, glandular epithelial cell; DSC, decidual stromal cell; iCTB, interstitial cytotrophoblast; dMΦ, decidual macrophage; dNK, decidual NK cell; Scale bars represent 50 μm. (**c**) Quantitative PCR showing Wnt5a mRNA expression in cell lines and primary cultures. Bars represent mean values ±SD of three different experiments performed in duplicates. Values of SGHPL-5 cells were arbitrarily set at 1 (calibrator). (**d**) Western blot analyses showing Wnt5a expression in protein lysates isolated villous (VSC) and decidual (DSC) stromal cells, primary cytotrophoblasts (CTBs, three different cell preparations), decidual NK cells (dNK), placental (pMΦ) and decidual (dMΦ) macrophages. Recombinant (rhu) Wnt5a and GAPDH were used as positive and loading control, respectively. (**e**) Representative western blot showing soluble Wnt5a in supernatants of decidual (DSC) and villous (VSC) stromal cells. Absolute protein concentration of Wnt5a, secreted in 24 hours, was determined by densitometrical scanning of western blot signals relative to 1 ng rhu Wnt5a. Bar graphs depict mean values ±SD of each three independent experiments/cell preparations. Wnt5a secretion was not significantly (n.s.) different between VSC and DSC. Furthermore, a representative western blot showing soluble Wnt5a in supernatants of differentiating DSC is shown. Cells were cultivated in the presence of 0.5 mM 8-Bromo-cAMP, or 10 nM estrogen (E)/1 μM progesterone (P) or both. After 6 days cells were counted and protein lysates of conditioned media were adjusted to cell numbers. Full-length western blots are shown in Supplementary Figure 3.

To gain first insights into the expression pattern of Wnt5a in first trimester placenta and decidua, tissue sections and purified cell populations were investigated by immunofluorescence, western blotting and RT-qPCR, respectively (Fig. 1). Wnt5a was detected in macrophages and villous stromal cells as well as in villous CTBs and the syncytium of placental sections (Fig. 1a). Immunofluorescence of decidual tissues revealed expression of the ligand in glandular epithelial cells, interstitial CTBs, stromal cells, macrophages and uterine natural killer (uNK) cells (Fig. 1b). Comparative gene expression profiling of us^33^ and others^34^ also suggested expression of Wnt5a mRNA in proliferative, epidermal growth factor receptor (EGFR)-positive CTBs and invasive, human leukocyte antigen-G-positive EVTs (not shown). However, Wnt5a transcript levels, measured by quantitative PCR, were low in first trimester CTBs compared to villous and decidual stromal cells, whereas JEG-3 did not express the ligand (Fig. 1c). Accordingly, analyses of cellular extracts indicated abundant Wnt5a protein levels in stromal cells of early pregnancy and trophoblastic SGHPL-5 cells whereas different primary CTB pools produced low amounts (Fig. 1d). Wnt5a was also detectable in purified placental and decidual CD14^+^ macrophages and in CD56^+^ decidual NK cells, but was absent from JEG-3 cells (Fig. 1d). Furthermore, Wnt5a was present in conditioned media of villous and decidual stromal cells (Fig. 1e). Quantification of western blots and adjustment to recombinant Wnt5a revealed that both stromal cell types released around 0.2 ng Wnt5a per μg cellular protein per 24 hours (Fig. 1e). Wnt5a secretion, however, was not affected upon *in vitro* differentiation of primary decidual stromal cells (Fig. 1e), which were cultivated in the presence of cAMP and/or estrogen/progesterone as previously mentioned^35^.

### Wnt5a promotes proliferation of villous and cell column cytotrophoblasts

**Figure 2:**
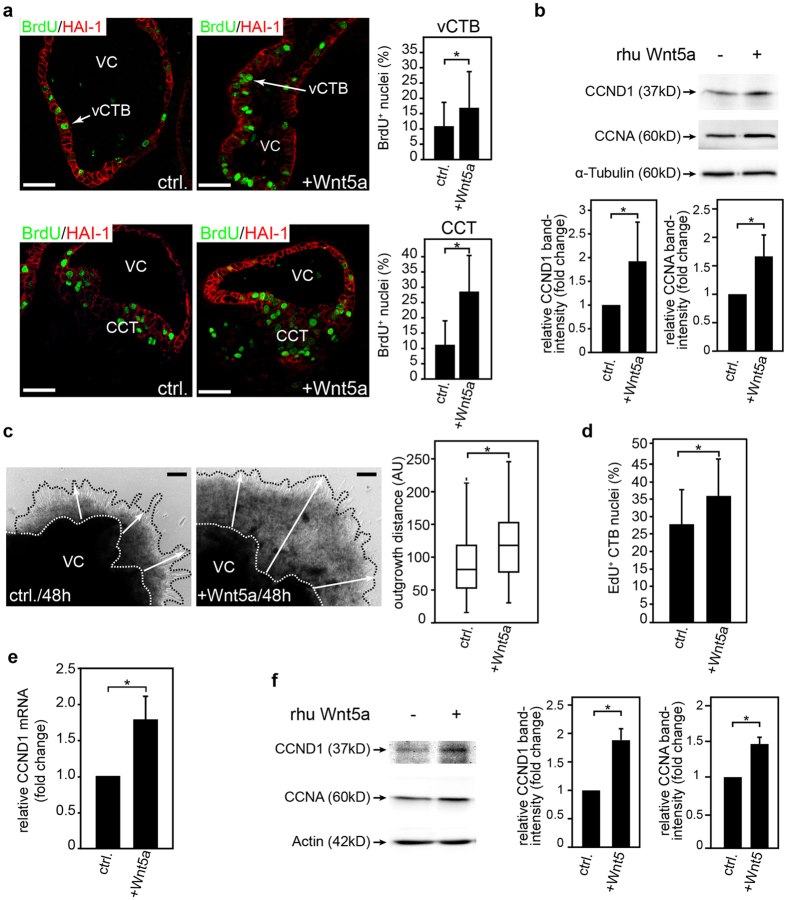
Wnt5a increases proliferation in first trimester villous explant cultures and primary cytotrophoblasts. (**a**) Wnt5a elevates BrdU labeling in floating explant cultures. Villous explants isolated from n = 12 placentae (7^th^–9^th^ week) were incubated in the absence (ctrl.) or presence of rhu Wnt5a (three different experiments). Representative pictures (scale bars 50 μm) showing BrdU labeling in villous cytotrophoblasts (vCTB, upper panel) and cell column trophoblasts (CCT, lower panel) after 24 hours of treatment. Hepatocyte growth factor activator inhibitor type 1 (HAI-1) was used to specifically mark cytotrophoblasts in placental sections. VC, villous core; vCTB, villous cytotrophoblast; CCT, cell column trophoblast; bar graphs represent mean values ± SD of the percentage of BrdU + villous CTBs and CCTs each. *p < 0.05; (**b**) Western blot analyses showing Wnt5a-stimulated (24 hours) expression of cyclin D1 (CCND1) and cyclin A (CCNA) in whole tissue lysates of floating explant cultures. A representative blot of 8^th^ week placentae is shown. Bar graphs depict mean values ± SD of CCND1 and CCNA signals measured in western blots of three different experiments performed with explants of 12 different placentae. For relative comparison values of untreated cultures were arbitrarily set at 1. *p < 0.05; (**c**) Wnt5a increases outgrowth of villous explants seeded on collagen I. Representative images (scale bar 50 μm) are depicted. Box blot shows median values of the outgrowth distance of Wnt5a-treated explants compared to controls (ctrl) measured in 4 different experiments using explants derived from 14 different placentae. *p < 0.05; (**d**) Rhu Wnt5a stimulates EdU incorporation (20 hours) into primary CTBs. Bar graph shows mean values ± SD of the percentage of EdU^ + ^CTBs of three different CTB preparations (n = 16 pooled placentae). *p < 0.05; (**e**) Quantitative real-time PCR of cyclin D1 (CCND1) transcript levels in untreated (ctrl.) and Wnt5a-treated (24 hours) primary CTBs. Mean values ± SD show CCND1 mRNA normalized to TBP expression of three experiments/CTB isolation (n = 13 pooled placentae). *p < 0.05; (**f**) Western blot analyses detecting protein expression of CCND1 and CCNA in Wnt5a-stimulated primary CTBs. A representative western blot is shown. Bar graphs show mean values ± SD of CCND1 and CCNA signals measured in each three independent experiments (CTBs isolated from 11 different placentae). For relative comparison values of untreated cultures were arbitrarily set at 1. *p < 0.05. Full-length western blots are shown in Supplementary Figure 3.

First trimester villous explant cultures and primary CTBs were treated with recombinant human (rhu) Wnt5a to assess the role of the ligand in trophoblast proliferation (Fig. 2). Immunofluorescence analyses revealed that Wnt5a increased BrdU labeling of villous CTBs and cell column trophoblasts (CCTs) in floating villous explants (Fig. 2a). Wnt5a also stimulated cyclin D1 and cyclin A protein expression in the latter (Fig. 2b). Accordingly, incubation with rhu Wnt5a increased the outgrowth distance of collagen I-seeded villous explants (Fig. 2c). Similarly, purified CTBs displayed elevated EdU incorporation (Fig. 2d) and cyclin D1 mRNA expression (Fig. 2e) in the presence of the particular Wnt ligand. Moreover, Wnt5a increased protein expression of cyclin D1 and cyclin A in these cultures (Fig. 2f). In contrast to its positive effects on trophoblast proliferation, rhu Wnt5a did not alter migration of primary CTBs through fibronectin-coated transwells (Supplementary Figure 1).

### Gene silencing of Wnt5a decreases proliferation of trophoblastic SGHPL-5 cells

**Figure 3:**
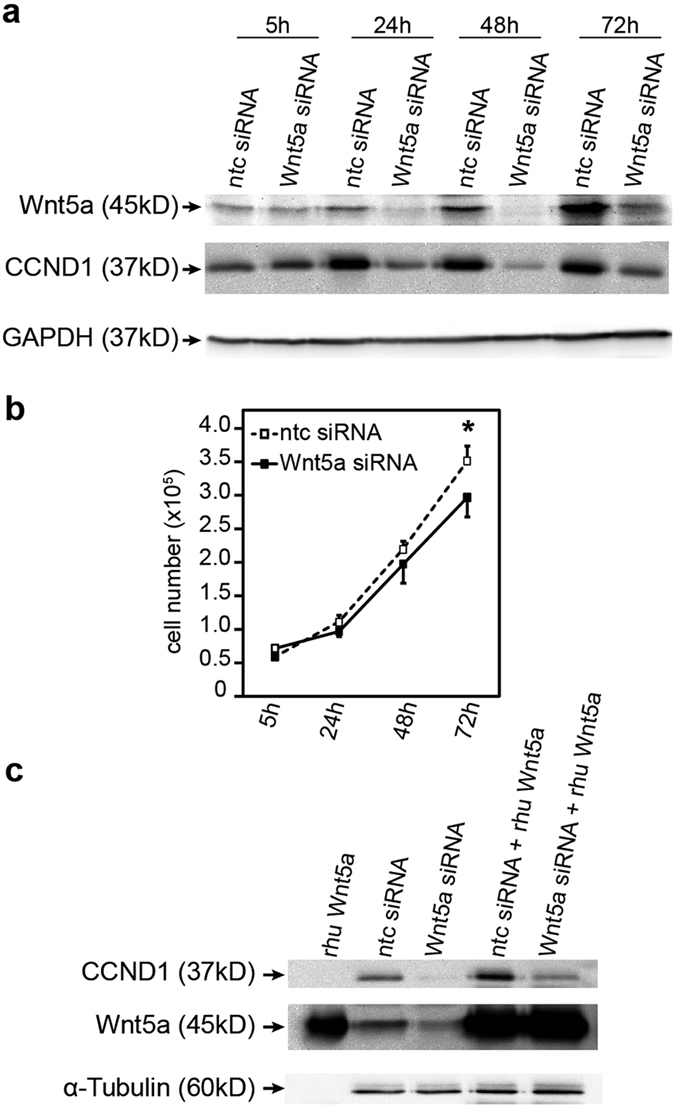
Gene silencing of Wnt5a reduces SGHPL-5 cell proliferation and cyclin D1 expression. (**a**) Representative western blot showing Wnt5a and cyclin D1 (CCND1) expression in protein lysates isolated from SGHPL-5 cells treated with Wnt5a siRNAs or non-targeting controls (ntc) for 5, 24, 48 and 72 hours. As a loading control GAPDH was used. (**b**) Proliferation of SGHPL-5 cells in the presence of Wnt5a siRNAs or non-targeting (ntc) controls. Graph, showing cumulative cell numbers at different time points, represents mean values ± SD of three different experiments performed in duplicates. *p < 0.05; (**c**) Cells were pre-treated with siRNAs against Wnt5a or non-targeting control (ntc) for 24 hours before rhu Wnt5a was added for additional 24 hours. Expression of Wnt5a and cyclin D1 (CCND1) were analysed by western blotting. Rhu Wnt5a and α-tubulin were used as positive and loading controls, respectively. A representative example out of three is shown. Full-length western blots are shown in Supplementary Figure 3.

Addition of rhu Wnt5a to trophoblastic SGHPL-5 cells did not change their proliferation rate (data not shown). Since SGHPL-5 cells express high endogenous Wnt5a levels, we speculated that growth of these cells cannot be further increased by adding exogenous Wnt5a. Therefore, the effects of siRNA-mediated Wnt5a silencing on SGHPL-5 cell proliferation and cyclin D1 expressions were investigated (Fig. 3). Western blot analyses of SGHPL-5 cell extracts indicated that downregulation of Wnt5a decreased cyclin D1 expression in a time-dependent manner (Fig. 3a). Evaluation of cumulative cell numbers suggested that Wnt5a gene-silenced SGHPL-5 cells grew less efficiently (Fig. 3b). Furthermore, supplementation of rhu Wnt5a to Wnt5a siRNA-treated cultures restored cyclin D1 expression (Fig. 3c). In contrast to SGHPL-5 cells, gene silencing of Wnt5a in CTBs did not change their basal proliferation rate (data not shown).

### Wnt 5a-induced proliferation of cytotrophoblasts involves MAPK signalling

**Figure 4:**
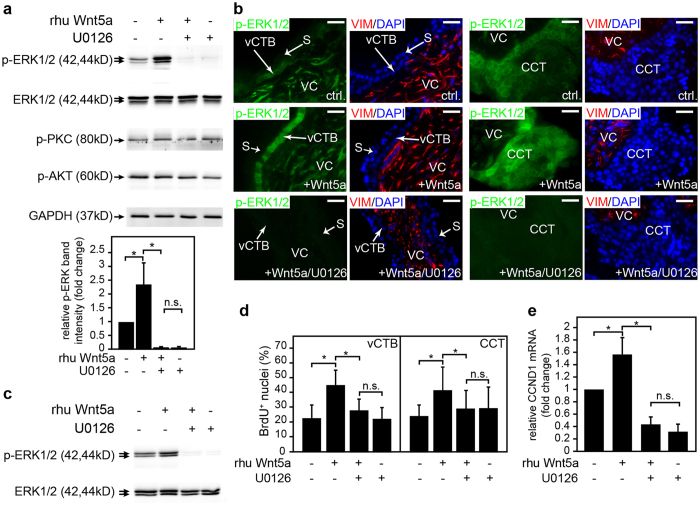
Wnt5a-induced proliferation of primary trophoblast cultures involves activation of MAPK signalling. (**a**) Wnt5a increases MAPK phosphorylation in first trimester primary CTBs. Isolated CTBs were pre-treated with or without U0126 before rhu Wnt5a was added for 20 minutes. Phosphorylation of p44/42 ERK1/2 (Thr202/Tyr204), proteinkinase C (PKC; Ser660) and AKT (Ser473) were analysed by western blotting. Representative examples are shown. Non-phosphorylated ERK1/2 and GAPDH were used as loading controls. Bar graphs show mean values ± SD of the combined p-ERK1/2 signals (normalized to GAPDH) obtained from western blots of three independent CTB preparations (n = 16 placentae). For relative comparison values of untreated cultures were arbitrarily set at 1. *p < 0.05; (**b**) Wnt5a promotes MAPK phosphorylation (p-ERK1/2) in villous cytotrophoblasts (vCTBs) and cell column trophoblasts (CCTs) of floating villous explants, prepared from - 7^th^ week placentae. After incubation in the absence or presence of U0126 for 30 minutes, explants were incubated with rhu Wnt5a for additional 1.5 hours. ERK1/2 phosphorylation was detected by immunofluorescence. Co-staining with vimentin was used to mark the villous core (VC), slides were counterstained with DAPI. Representative images (scale bar 50 μm) are depicted. vCTB, villous cytotrophoblast; CCT, cell column trophoblast; S, syncytium. (**c**) Western blot analysis showing Wnt5a-stimulated activation of p44/42 MAPK (ERK1/2) in whole tissue lysates of floating explant cultures. A representative blot obtained from explants of 8^th^ week placentae is shown. Total ERK1/2 was used as loading control. (**d**) BrdU labeling of floating villous explant cultures in the absence or presence (24 hours) of Wnt5a and/or U0126. Labeling of cultures and counting of the BrdU/DAPI ratio in vCTBs and CCTs, respectively, were performed as described above. Bar graphs represent mean values ± SD of the percentage of BrdU^+^ villous CTBs and CCTs each of three independent experiments performed with explants isolated from 12 different placentae. Controls were derived from 7 different experiments (explants isolated from 32 different placentae). *p < 0.05; ns, not significant; (**e**) Quantitative real-time PCR showing relative CCDN1 expression normalized to TBP in CTBs treated with Wnt5a and/or U0126 for 24 hours. Bars represent mean values ± SD of each three different cell preparations performed in duplicates. Values of untreated cultures were arbitrarily set at 1. *p < 0.05; ns, not significant; Full-length western blots are shown in Supplementary Figure 3.

To determine non-canonical downstream effects of Wnt5a in primary trophoblast cultures, activation of different signalling cascades was evaluated (Fig. 4). Western blot analyses of primary CTBs suggested that rhu Wnt5a induced phosphorylation of MAPK (p42/44) which could be specifically blocked in the presence of the chemical inhibitor U0126 (Fig. 4a). In contrast to ERK1/2, activation of protein kinase C (PKC) or AKT could not be observed. Accordingly, Wnt5a-stimulated MAPK phosphorylation was detected in villous CTBs and CCTs of floating villous explant cultures using immunofluorescence, whereas supplementation of U0126 inhibited ligand-induced activation of the enzyme (Fig. 4b). These data were confirmed by western blotting of whole tissue lysates isolated from the placental explants (Fig. 4c). In contrast to U0126, addition of recombinant Dkk1 did not block Wnt5a-induced ERK activation, reassuring the role of Wnt5a in non-canonical Wnt signalling of CTBs (Supplementary Figure 2). To prove that MAPK signalling is involved in Wnt5a-stimulated trophoblast proliferation, BrdU incorporation into villous CTBs and CCTs of explant cultures was studied in the absence or presence of Wnt5a and/or U0126 (Fig. 4d). Determination of the BrdU/DAPI ratio revealed that Wnt5a-induced proliferation of both vCTB and CCTs was inhibited in the presence of the chemical inhibitor. Moreover, U0126 also blocked Wnt5a-induced cyclin D1 mRNA expression in cultured CTBs (Fig. 4e).

### Wnt 5a suppresses camptothecin-induced apoptosis

**Figure 5:**
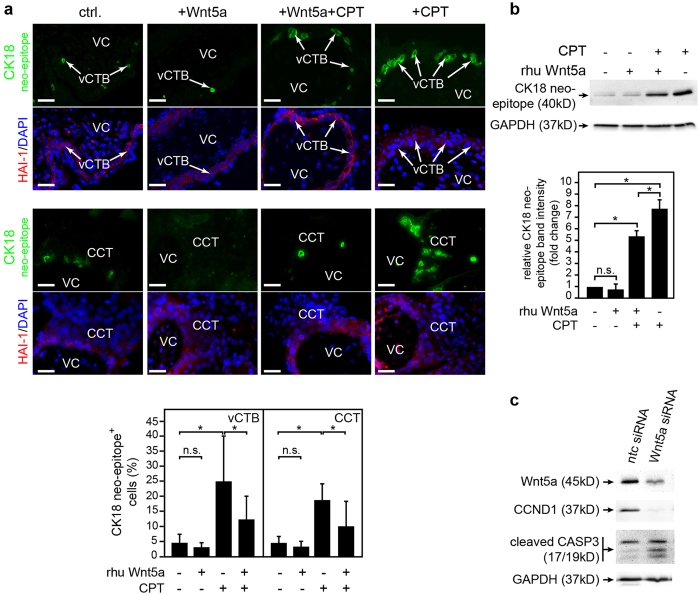
Wnt5a suppresses camptothecin-induced apoptosis. Preparation of primary cultures, immunofluorescence and western blotting were performed as described in Materials and Methods. (**a**) Wnt5a inhibits cytokeratin 18 (CK18) neo-epitope expression in floating villous explants. Cultures were treated with rhu Wnt5a for 24 hours in the absence or presence of camptothecin. Expression of caspase-cleaved CK18 neo-epitope was analysed by immunofluorescence in the different cytotrophoblast populations using M30 cytoDEATH antibodies. Co-stainings with HAI-1 were used to mark villous cytotrophoblasts (vCTBs) and cell column trophoblasts (CCTs). Nuclei were stained with DAPI. VC, villous core; Representative examples (scale bar 50 μm) of 8^th^ week placentae are shown. Mean values (3 experiments performed with explants derived from 11 different placentae) in the bar graph represent the percentage ± SD of CK18 neo-epitope-labelled vCTBs and CCTs under the different treatments. *p < 0.05; ns, not significant; (**b**) Western blot showing CK18 neo-epitope expression in primary CTBs. Cells were pre-treated with rhuWnt5a and subsequently incubated with camptothecin for 24 hours. A representative blot obtained from protein lysates of a 12^th^ week CTB preparation is shown. The relative signal intensity was determined by densitometrical scanning and quantification of bands. Bar graphs show mean values ± SD normalized to GAPDH obtained from western blots of three different CTB preparations (n = 19 pooled placentae). Untreated control was arbitrarily set to 1. *p < 0.05; ns, not significant; (**c**) Western blot illustrating induction of active caspase-3 (CASP3) upon siRNA-mediated gene silencing (48 hours) of Wnt5a in SGHPL-5 cells. As demonstrated above downregulation of Wnt5a also decreased cyclin D1 (CCND1) expression in these cells. A representative blot out of three is shown. Full-length western blots are shown in Supplementary Figure 3.

The effects of Wnt5a treatment or siRNA-mediated downregulation of the ligand on trophoblast survival were analysed in villous explant cultures, primary CTBs and SGHPL-5 cells, respectively (Fig. 5). In floating explants rhu Wnt5a diminished camptothecin-induced expression of caspase-cleaved, cytokeratin 18 (CK18) neo-epitope, both in vCTBs and CCTs (Fig. 5a). Basal apoptosis was not significantly affected by Wnt5a in these cultures, although a trend towards lower levels could be observed. Similarly, abundance of the particular apoptotic marker decreased in campthotecin-treated primary CTBs when rhu Wnt5a was added (Fig. 5b). Accordingly, siRNA-induced gene silencing of Wnt5a in SGHPL-5 cells increased expression of cleaved caspase-3 (Fig. 5c).

### Stromal cell-derived Wnt5a promotes p42/44 MAPK phosphorylation and proliferation of primary cytotrophoblasts

**Figure 6:**
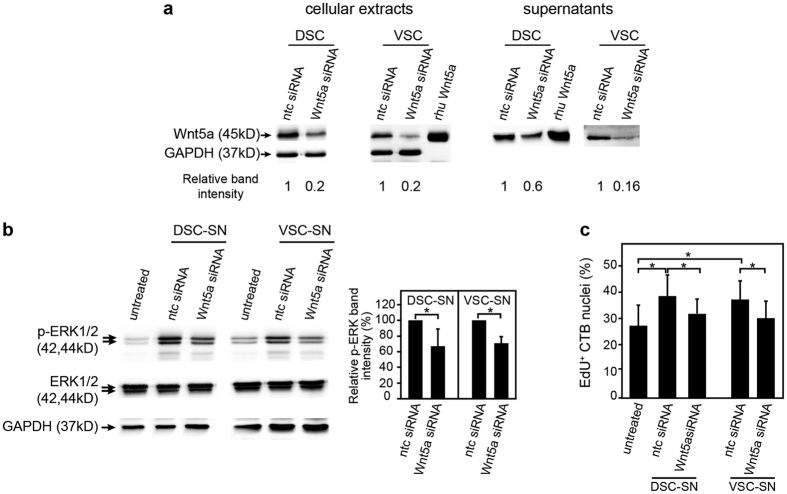
Decidual and villous stromal cell-derived Wnt5a promotes MAPK phosphorylation and proliferation of primary CTBs. Isolation, cultivation and gene silencing of Wnt5a in primary villous (VSC) and decidual stromal cells (DSC) were performed as described in Materials and Methods. Isolated CTBs were stimulated with diluted supernatants (SN) aspirated from the siRNA-treated stromal cell cultures. MAPK phosphorylation and proliferation of CTBs were analysed by western blotting and EdU labeling, respectively; ntc, non-targeting control. (**a**) Representative western blots showing siRNA-mediated downregulation of Wnt5a in cellular extracts and supernatants of decidual and villous stromal cells. Rhu Wnt5a and GAPDH were used as positive and loading control, respectively. Wnt5a signals (ntc set to 1) were normalised to GAPDH (cellular extracts) or total cellular protein content (supernatants). (**b**) Western blots showing activated and total p42/44 MAPK in primary CTBs after incubation with conditioned medium from control (ntc) and Wnt5a siRNA-treated villous or decidual stromal cells. Representative examples are shown. Bar graph represents mean values ± SD of p-ERK signals (ERK1 plus ERK2) normalized to GAPDH obtained from three independent CTB preparations (ntc set to 100%). (**c**) EdU labeling of primary CTBs after incubation with supernatants of ntc or Wnt5a siRNA-treated villous or decidual stromal cells. Bar graphs (3 experiments/CTB preparations performed with 13 pooled placentae) represent mean values ± SD of the percentage of EdU^+^ primary CTBs normalized to total number of DAPI-positive nuclei. *p < 0.05; Full-length western blots are shown in Supplementary Figure 3.

To assess whether Wnt5a, secreted from decidual and villous stromal cells, exerts similar effects as the recombinant ligand, primary CTBs were incubated with supernatants obtained from Wnt5a gene-silenced cultures (Fig. 6). Western blot analyses revealed that the siRNA-treatment decreased Wnt5a protein expression in cellular extracts and supernatants of decidual and villous stromal cells, respectively (Fig. 6a). Compared to non-targeting controls, activation of p42/44 MAPK was significantly diminished when primary CTBs were stimulated with conditioned medium from Wnt5a siRNA-treated stromal cell cultures (Fig. 6b). Accordingly, BrdU labelling of CTBs was also less efficient upon incubation with supernatants from Wnt5a gene-silenced villous or decidual stromal cells (Fig. 6c).

## Discussion

Signalling through Wnt5a is critical for development and tumorigenesis^36^. During gastrulation Wnt5a is expressed in a gradient at the caudal end of the murine embryo at a time when growth and patterning of the embryonic A-P axis takes place^37^. Around E6.5-9.5 abundant levels of Wnt5a were detectable in different mesodermal compartments, for example in cells of the primitive streak and allantois, and later on in outgrowing embryonic structures such as limbs, genitals and face^38^. Homozygous mutation of Wnt5a in mice provoked truncation of tail, limbs and snout amongst other defects suggesting that the ligand is required for induction and proliferation of different mesodermal stem cells and progenitors^38^. In the murine endometrium, Wnt5a is also predominantly expressed in stromal cells suggesting that Wnt5a-induced mesenchymal-epithelial interactions could be crucial for proper gland formation^25^,^39^. Indeed, Wnt5a was shown to be essential for morphogenesis of the posterior female reproductive tract as well as postnatal growth of uterine glands^25^. Moreover, loss or overexpression of Wnt5a in the murine uterus impairs implantation, decidualization and placental branching morphogenesis indicating that regulated levels of the ligand are critical for a successful pregnancy^26^.

In the human reproductive tract, Wnt5a mRNA was detected in cultured endometrial epithelial and stromal cells and its expression did not change between proliferative and secretory phase of the menstrual cycle^23^,^24^. Moreover, Wnt5a protein expression was observed in isolated first and third trimester placental trophoblasts^22^. Therefore, the descriptive data suggested that, similar to its role in mice, human Wnt5a could also be a critical regulator of endometrial and placental development and function. To gain first insights into the putative role of Wnt5a in human placentation we herein analysed localization of the particular ligand at the fetal-maternal interface and investigated its biological role in trophoblasts. Immunofluorescence of first trimester placental and decidual tissues revealed that Wnt5a is abundant in the decidual stroma and in the core of placental villi. Accordingly, primary villous and decidual stromal cells secreted high levels of Wnt5a, whereas cultivated CTBs produced low amounts. Moreover, the ligand was also present in villous and decidual macrophages, uterine NK cells and decidual glands. Hence, Wnt5a could have multiple functions in placenta and maternal decidua involving autocrine as well as paracrine actions. Previously, macrophage-specific Wnt5a expression has been noticed in tumor-associated macrophages and upon bacterial infection which suggested a role of the ligand in inflammatory responses^40^,^41^. However, more recent data showed that Wnt5a induces an immunosuppressive macrophage phenotype and inhibits differentiation of monocytes into inflammatory M1 macrophages^42^. Noteworthy, alternatively activated M2-like macrophages, which are thought to confer immunogenic tolerance to the semi-allogeneic fetus^43^, increase in the decidua from first to second trimester^44^. Therefore, we speculate that Wnt5a, secreted from monocytes and stromal cells, could affect decidual macrophages thereby contributing to the generation of an immune tolerant milieu required for successful progression of pregnancy. Moreover in analogy to other developing tissues and organs, Wnt5a could play a pivotal role at the fetal-maternal interface. For example, Wnt5a has been implicated in angiogenesis promoting proliferation, migration and survival of endothelial cells and also controls differentiation of mesenchymal stem cells into different lineages^31^. Although we observed abundant levels of Wnt5a in primary decidual stromal cells, its expression was not regulated upon *in vitro* differentiation of these cultures with cAMP and estrogen/progesterone. However, proliferation of Wnt5a siRNA-treated decidual fibroblasts was slightly decreased after 5 days of cultivation (not shown), suggesting a weak autocrine effect of the ligand on uterine stromal cell growth.

According to its role in progenitor proliferation and mesenchymal-epithelial interactions in mice^25^,^38^, stimulation of the villous trophoblast epithelium could be a main function of stromal-cell derived Wnt5a. Whereas the migratory capacity of primary CTBs was not affected by the ligand, proliferation of villous CTBs and CCTs in floating explant cultures increased in the presence of exogenous rhu Wnt5a. Moreover, EdU labeling of primary CTBs was significantly enhanced. Compared to controls, conditioned media of Wnt5a gene-silenced villous or decidual stromal cells stimulated CTB growth less efficiently. Therefore, we speculate that decidua-derived Wnt5a could be a critical soluble factor promoting proliferation of proximal CCTs during the first trimester of pregnancy. On the other hand, mitotic activity of villous CTBs and proximal CCTs could be stimulated by Wnt5a released form the underlying placental mesenchyme. Autocrine effects of Wnt5a on trophoblast proliferation could not be detected, likely due to the fact that CTBs express low amounts of the ligand. In contrast, Wnt5a was abundant in the trophoblastic cell line SGHPL-5 and silencing of its mRNA consequently impaired proliferation. Interestingly, the decidual glands also express Wnt5a suggesting that glandular secretion of the factor could support trophoblast growth and placental branching morphogenesis during the period of histiotrophic nutrition and early placental development. Along those lines, the placental spongiotrophoblast layer of mice with uterine loss of Wnt5a was smaller at E12 compared to wildtype animals corroborating a critical role of the ligand in trophoblast proliferation and/or survival^26^. Non-canonical Wnt signalling through Wnt5a is complex and involves numerous pathways and receptors^28^,^31^. In the planar cell polarity (PCP)/convergent extension pathway Wnt5a was shown to promote cell movements during gastrulation involving activation of RhoA/Rho kinase and Rac/c-Jun terminal kinase^31^. Wnt5a can also increase intracellular Ca^2+^ levels by release from internal stores thereby activating protein kinase C and calcium/calmodulin-dependent protein kinase II^29^. The particular pathway, which is also elicited by Wnt11, controls cell fate decisions and differentiation^29^. However, it is unlikely that Wnt5a triggers Wnt/Ca^2+^ signalling in human CTBs since activation of protein kinase C could not be observed. Moreover, Wnt5a can also induce signalling through PI3K/AKT, MAPK or nuclear factor κB (NFκB) controlling many different cellular functions such as proliferation, differentiation, migration or inflammation^30^,^32^. Whereas the ligand did not affect AKT, phosphorylation of p42/44 MAPK (ERK1/2) was noticed in Wnt5a-treated villous explant cultures and primary CTBs. Chemical inhibition with U0126 did not only block paracrine activation of the enzymes by exogenously added Wnt5a, but also reduced ligand-induced proliferation of vCTBs and CCTs in explant cultures as well as cyclin D1 expression. Therefore, the data suggest that ERK1/2 are critical for the Wnt5a-stimulated mitotic activity of placental trophoblasts.

Besides controlling CTB proliferation Wnt5a could also promote trophoblast survival since treatment with the recombinant ligand inhibited camptothecin-induced apoptosis. Wnt5a-dependent survival involves the expression of pro-survival genes in other cellular systems, mediated by signalling through protein kinase A/cAMP response element-binding protein (CREB), PI3K/Akt, NFκB or p42/44 MAPK^30^,^45^,^46^. Since the latter pathway triggers CTB proliferation, we speculate that it could also play a role in inhibiting trophoblast apoptosis. In contrast to primary CTBs, the function of Wnt5a is altered in tumorigenic trophoblasts since treatment of JAR cells with the ligand increased apoptosis and suppressed proliferation^47^. These data support the common view that Wnt5a functions highly depend on the cellular context and the specific expression patterns of its receptors.

Indeed, Wnt5a may exert its effects on trophoblasts through different receptor complexes. Wnt5a was shown to activate several different Fzd members, i.e. Fzd2, Fzd3, Fzd4, Fzd5 and Fzd8 in a canonical or non-canonical fashion, of which Fzd5 is the most abundant receptor in first trimester CTBs^22^. In mice, homozygous mutation of Fzd5 affected angiogenesis in the placenta and yolk sac^48^. Moreover, a recent study indicated that Wnt signalling through Fzd5 is essential for placental morphogenesis promoting maintenance of glial cells missing 1 (GCM1) expression at distinct branching sites^49^. Interestingly, gene expression profiles published by us and others^33^,^34^ suggested that Fzd5 mRNA is strongly expressed in non-invasive CTBs but largely absent from EVTs (not shown). The differential expression of Fzd5 might explain why EVT migration could not be stimulated by Wnt5a. On the other hand, Wnt5a also signals through the receptor tyrosine kinase Ror2 which can bind the ligand on its own or act as Fzd co-receptor^36^. Of note, Wnt5a-Ror2 signalling was shown to provoke activation of p24/44 MAPK^50^, which lets us speculate that this pathway could also play a role in CTB proliferation. Moreover, since transactivation of the EGFR by Wnt5a has been noticed^51^,^52^, it is possible that the ligand also stimulates p42/44 MAPK phosphorylation through EGFR-Ras-Raf-MEK-ERK signalling. Further investigations are needed to clarify the full network of Wnt5a receptors and their downstream signalling cascades in first trimester CTBs.

**Figure 7:**
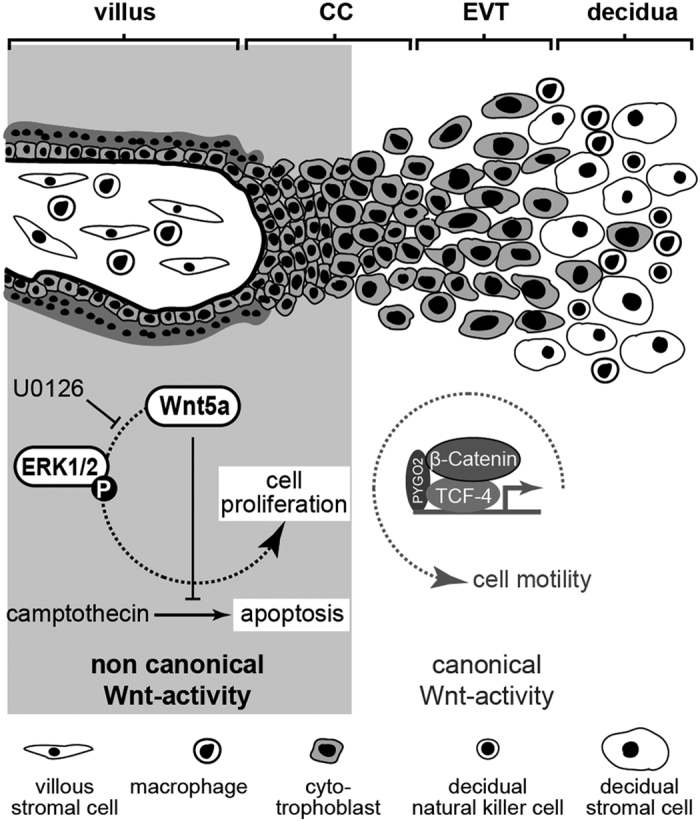
Model system describing the presumptive role of non-canonical Wnt5a signalling in first trimester CTBs. Whereas activation of canonical, TCF-4/β-catenin-mediated Wnt signalling is associated with migration, EVT marker gene expression and differentiation^19^,^20^, non-canonical, Wnt5a-induced signalling promotes proliferation and survival of CTBs and CCTs through p42/44 MAPK. Wnt5a could be largely derived from decidual and villous stromal cells exerting paracrine effects on CCTs and villous CTBs.

In summary, the present data suggest that Wnt5a, secreted by different decidual and placental cell types, could be a critical regulator of human placentation and trophoblast function (Fig. 7). Besides controlling proliferation though p42/44 MAPK activation, it also promotes survival of CTBs. Moreover, the pivotal role of Wnt5a during reproductive development and its abundance at the fetal-maternal interface suggest that the ligand could eventually regulate numerous processes in human placenta and decidua such as angiogenesis, stromal cell growth, macrophage differentiation and immunomodulation.

## Materials and Methods

### Tissue collection

Placental and decidual tissues (6–13 weeks of gestation, n = 88) were obtained from elective pregnancy terminations. Utilization of tissues and all experimental procedures were approved by the ethical committee of the Medical University of Vienna. Methods were carried out in accordance with the approved guidelines. Written informed consent was obtained from all subjects.

### Cultivation of SGHPL-5 cells

SGHPL-5 cells were cultivated in DMEM/Ham’s F-12 (1:1) (Gibco BRL Life Technologies, Paisley, UK) supplemented with 0.05 mg/ml gentamicin (Gibco) and 10% fetal calf serum (FCS) (Biochrom AG, Berlin, Germany) as mentioned^53^.

### Cultivation of placental primary cells

First trimester CTBs were isolated by Percoll gradient centrifugation from pooled placentae (n = 4–7) as described^54^,^55^ and plated (40 minutes) in culture medium (DMEM/Ham’s F-12/10% FCS/0.05 mg/ml gentamicin/0.5 μg/ml fungizone, Gibco) allowing for adherence of contaminating stromal cells. CTBs were seeded onto fibronectin-coated dishes (2.5 × 10^5^ cells/cm^2^) in culture medium and characterised by immunofluorescence using cytokeratin 7 (KRT7, OV-TL12/30, 1.96 μg/ml; Dako, Glostrup, Denmark) and vimentin (VIM; SP20, Abcam, 1:100) antibodies. Alexa Fluor 488 (goat anti-mouse) and Alexa Fluor 568 (goat anti-rabbit) were used as secondary antibodies (2 μg/ml, Molecular Probes, Invitrogen), respectively. Vimentin-positive cells were less than 1%.

To isolate placental macrophages, villi were digested twice in 0.125% trypsin (Gibco)/0.5 mg/ml DNAse I (Sigma Aldrich)/25 mM HEPES diluted in 1xHanks’ balanced salt solution under agitation for 15 min at 37 °C and enriched by Percoll gradient centrifugation. Macrophages were purified by positive selection using anti CD14 MicroBeads (Miltenyi Biotec, Bergisch Gladbach, Germany).

Villous stromal cells were cultivated by seeding trypsin digested and Percoll gradient centrifugation enriched placental villi as described onto plastic cell culture dishes for 40 minutes^54^,^55^. Non adhering cells were removed and cell culture dishes were overlaid with fresh culture medium. As soon as cells reached confluency they were split and used for further experiments.

### Cultivation of decidual primary cells

Decidua basalis tissues (7–12 week n = 6) were used to isolate stromal and uNK cells as mentioned^56^,^57^. Briefly, small pieces of tissues (3 mm^2^) were digested 3 times for 20 minutes under agitation using collagenase I (2 mg/ml, Gibco) and DNAse I (0.5 mg/ml Sigma). For cultivation of decidual fibroblasts cells were filtered over a 70 μm cell strainer and seeded in the above mentioned cell culture medium. For *in vitro* decidualization cells were treated with of cAMP and estrogen/progesterone^35^. Decidual NK cells were isolated from the non-adherent cell population of plated decidual stromal cells by positive selection with CD56-PE antibodies and Anti-PE MicroBeads (MACS Miltenyi Biotec) 20 hours after plating. Decidual macrophages were purified using anti CD14 MicroBeads, directly after digestion of tissues.

### Immunofluorescence

First trimester placental and decidual tissues (6^th^–12^th^ week, n = 15) were fixed in paraformaldehyde and embedded in paraffin. Serial sections (3 μm) were analysed by immunofluorescence as described^58^. Primary antibodies were as follows: Wnt-ligand 5a (Wnt5a; 3A4; LSBio; 1:300), Vimentin (Vim 3B4, Dako, 1:100), Cytokeratin wide spectrum (PanKRT) (GeneTex; 1:100), Cytokeratin 7 and Vimentin (both mentioned above), CD14 (Atlas Antibodies, 1:200), CD56 (123C3, Cell Signaling, 1:50), Bromodeoxyuridine (BrdU; Bu20a, Dako, 26 μg/ml), hepatocyte growth factor activator inhibitor 1 (HAI-1; H-108, Santa Cruz, 1:100), cytokeratin 18 neo-epitope (M30 CytoDEATH, Roche, 1:100) and phospho-p44/p42 MAPK (Erk1/2) (Thr202/Tyr204) (D13.14.4E XP, Cell Signaling 1:200). Samples were incubated for one hour with 2 μg/ml of secondary antibody Alexa Fluor 488 (goat anti-mouse), Alexa Fluor 488 (goat anti-rabbit), Alexa Fluor 546 (goat anti-mouse) or Alexa Fluor 568 (goat anti-rabbit). Nuclei were stained with DAPI (1 μg/ml, Roche, Mannheim, Germany). Slides were analysed by fluorescence microscopy (Olympus BX50, Cell^P software) and digitally photographed.

### siRNA-mediated gene silencing

Gene silencing using a mixture of 4 different siRNAs targeting Wnt5a or a non-targeting (ntc) control pool (L-003939-00-0005 and D-001810-10-20 ON-TARGETplus SMARTpools, Dharmacon-Thermo Fisher Scientific, MA) was performed as published^59^. Wnt5a-gene silenced SGHPL-5 cells were used 5, 24, 48 or 72 hours post siRNA delivery. Primary villous and decidual stromal cells were passaged once after isolation before siRNAs were added. Cellular extracts and supernatants were collected 5 days after treatment.

### Quantitative PCR

RNA was isolated with PeqGold Trifast (Peqlab, Wilmington, DE, USA). Reverse transcription and quantitative real-time PCR (in duplicates) were performed using the 7500 Fast Real-time PCR system (Applied Biosytems) and TaqMan Gene Expression Assays CCND1 (ABI # Hs00277039_m1) and Wnt5a (ABI #Hs00998537_m1) as described^35^. 1 μl cDNA, 0.5 μl primers and 5 μl Fast 2x PCR-Mix (TaqMan Universal PCR Master Mix) were used per sample. Signals (ΔCt) were normalized to TATA-box binding protein (TBP, ABI # 4333769F). Relative expression levels were determined by using values of controls as a calibrator (ΔΔCt).

### Western blotting

Protein extracts were separated on SDS/PAA gels, transferred onto Amersham Hybond-P PVDF membranes and incubated with primary antibodies as mentioned^19^. The following antibodies were used: Wnt5a (C27E8, Cell Signaling, 1:1000), GAPDH (14C10, Cell Signaling 1:5000), α-tubulin (Calbiochem DM1A, 1:5000), actin (Sigma 1:2500) CCNA (clone 6E6, Thermo Scientific, 1:100), CCD1 (DSC-6 Santa Cruz 1:500) CD14 (Atlas Antibodies, HPA001887, 1:200) CD56 (123C3, Cell Signaling 1:1000), phospho-p44/p42 MAPK (ERK1/2) (Thr202/Tyr204) (D13.14.4E XP, Cell Signaling, 1:1000), p44/42 MAPK (ERK1/2) (Cell Signaling, 1:1000), phospho-PKC (pan) (βII Ser660) (Cell Signaling, 1:1000), phospho-AKT (Ser473) (D9E XP, Cell Signaling 1:1000), caspase-cleaved cytokeratin 18 neo-epitope (M30 CytoDEATH, Roche, 1:500), cleaved caspase-3 (Asp175) (5A1E, Cell Signaling, 1:1000). Subsequently, membranes were incubated for 1 hour with HRP-conjugated secondary antibodies (goat anti-mouse, Sigma, 1:50.000, or anti-rabbit, Cell Signaling, 1:5000). Signals were developed using ECL prime detection Kit (GE Healthcare) and visualized with FluorChemQ imaging system (Alpha Innotech, San Leandro, USA). Quantification was performed by Image J software.

### Proliferation assays

5 × 10^4^ SGHPL-5 cells, seeded in duplicates, were treated with ntc or Wnt5a siRNAs. Cell numbers were counted at 5, 24, 48 and 72 hours using Casy cell counting system (Casy1 Model TTC, Schärf System). Proliferation of primary CTBs was analysed by EdU Cell Proliferation assay EdU-Click 488 (base click, Tutzing, Germany). Cells were seeded onto fibronectin-coated 48 wells (duplicates), treated with rhu Wnt5a (500 ng/ml, R&D Minneapolis, USA) or supernatants of decidual or villous stromal cells (diluted 1:2 in cell culture medium), and 1 μM EdU (5-ethynyl-2′-deoxyuridine) was added for 20 hours. After fixation colour development was performed and CTBs were stained with cytokeratin 7 antibodies as mentioned above. Five immunofluorescence pictures were taken per condition using an EVOS FL Color Imaging System (100 fold magnification). Proliferation was quantified as the percentage of EdU positive-nuclei of KRT7-positive CTBs. Moreover, 12–15 villous explants of different placentae (7–9^th^ week) were cut into equivalent pieces and separated into treatment groups, incubated with vehicle (0.1% bovine serum albumin) or rhu Wnt5a overnight. Bromodeoxyuridine (BrdU, Roche, 1 μM) was added for additional 4 hours. After fixation and embedding, BrdU incorporation was detected by immunofluorescence. Proliferation was quantified as the percentage of BrdU-labelled nuclei of HAI-1-positive CTBs. 1500–2500 CTBs and CCT nuclei were evaluated. To investigate the role of ERK1/2, villous explants were pre-treated with 10 μM U0126 before supplementation of Wnt5a and BrdU for 24 h.

### Outgrowth in villous explant cultures

16 villous explants of pooled placentae (n = 4–6; 7–9^th^ week) were dissected per condition and, seeded onto rat tail collagen I as mentioned^60^ and treated with rhu Wnt5a. Outgrowth area was digitally photographed using the EVOS FL Color Imaging System. Outgrowth distance was quantified after 48 h of cultivation by measuring the distance from the villous tip to the migratory front using Adobe Photoshop CS5 (3 measurements per explant).

### Signalling in primary CTBs and villous explant cultures

Primary trophoblasts were serum-starved (2 hours) followed by treatment with cell culture medium for 1 hour and incubation with 10 μM U0126 and/or rhu Dkk1 (1 μg/ml R&D) for 1 h. Rhu Wnt5a or 0.1% bovine serum albumin (control) were added for 20 minutes before protein extracts were prepared. Additionally, serum-starved CTBs were treated with culture medium for 3 hours and subsequently stimulated with Wnt5a-depleted stromal cell supernatants (diluted 1:2 in medium) for additional 20 minutes. To study ERK activation in floating villous explant cultures, 12–15 placental explants of pooled placentae (n = 3–6, 7–8^th^ week) and condition were dissected, incubated with 10 μM U0126 for 30 min and treated with rhu Wnt5a for 1.5 hours, followed by immunofluorescence or western blotting.

### Apoptosis assays

Fibronectin-seeded primary CTBs were treated with Wnt5a or 0.1% BSA for 30 minutes before 0.5 μM camptothecin (CPT, MP Biomedicals Santa Ana CA, USA) was added for 24 hours. Cells were lysed and analysed by western blotting detecting caspase-cleaved cytokeratin 18 (CK18 neo-epitope). Also, 12–15 explants of pooled placentae (n = 4–7; 7–8^th^ week) were incubated with rhu Wnt5a for 30 minutes before camptothecin (0.5 μM) was supplemented for 20 hours. Tissues were analysed by immunofluorescence using antibodies against caspase-cleaved cytokeratin 18 (CK18 neo-epitope), HAI-1 and DAPI as mentioned above. Apoptotic trophoblasts were quantified as the ratio of CK18 neo-epitope-positive nuclei to HAI-1 positive vCTBs and CCTs. 1500-2500 cells were counted. Additionally, apoptosis was evaluated in Wnt5a-gene silenced SGHPL-5 cells detecting cleaved caspase-3 in western blots.

### Migration assay

Migration of primary CTBs through fibronectin-coated transwells were performed in the presence of rhu Wnt5a or rhu EGF (25 ng/ml) as recently described^19^. Undersides of filters were stained with KRT7 antibodies and DAPI. After mounting KRT7-positive CTBs with DAPI-positive nuclei were counted. Five non-overlapping pictures of each membrane representing approximately 40–50% of the overall surface were taken and analysed using ImageJ.

### Statistical Analyses

Statistical analyses were performed with Student’s unpaired *t*-test or Mann-Whitney U test using SPSS 14 (SPSS Inc.). Gaussian distribution and equality of variances were examined with Kolmogorov-Smirnov test and Levene Test, respectively. For multiple comparisons One-Way ANOVA and appropriate post-hoc tests were performed. A *P* value of <0.05 was considered statistically significant.

## Additional Information

**How to cite this article**: Meinhardt, G. *et al.* Wingless ligand 5a is a critical regulator of placental growth and survival. *Sci. Rep.* **6**, 28127; doi: 10.1038/srep28127 (2016).

## Supplementary Material

Supplementary Information
