# Supplementary material for: Wingless ligand 5a is a critical regulator of placental growth and survival
Source: Sci Rep. 2016 Jun 17;6:28127. doi: 10.1038/srep28127 (PMC4911582; doi:10.1038/srep28127)

Supplementary Informations

Wingless ligand 5a is a critical regulator of placental growth and survival

Gudrun Meinhardt, Leila Saleh, Gerlinde R. Otti, Sandra Haider, Philipp Velicky, Christian Fiala,

Jürgen Pollheimer, Martin Knöfler

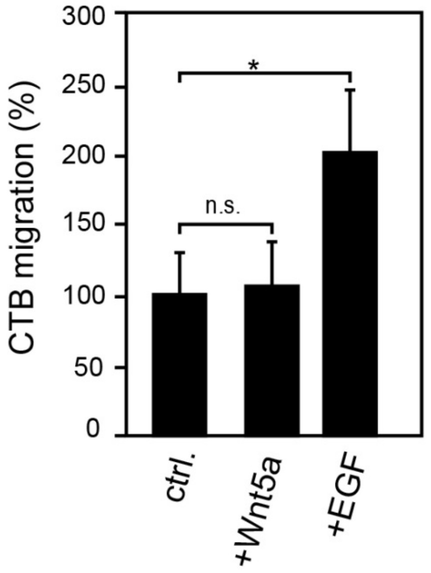

Supplementary Figure 1. Wnt5a does not affect migration of purified primary CTBs through fibronectin-coated transwells. Preparation of first trimester CTBs and migration assays (24 hours) in the absence (ctrl) or presence of rhu Wnt5a (500 ng/ml) were done as described in Methods. EGF (25 ng/ml) was used as a positive control. Bars indicate mean values ± SD of three different cell preparations analysed in duplicates. *, $p<0.05$; ns, not significant.

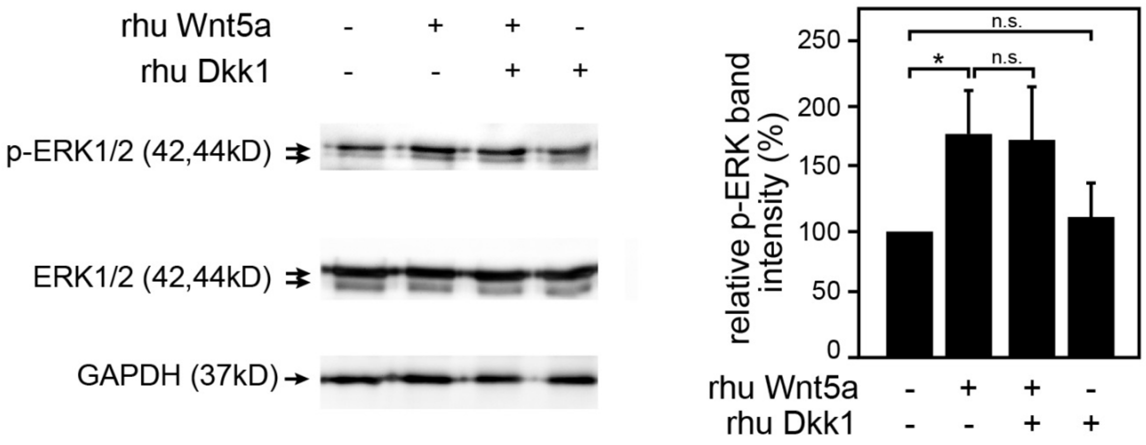

Supplementary Figure 2. Dkk1 does not affect Wnt5a-induced MAPK activation. Primary CTBs were incubated in the absence or presence of recombinant (rhu) Wnt5a and/or rhu Dkk1 (1µg/ml) and analysed by western blotting. Representative examples are shown. Bar graph represents mean values ± SD of combined p-ERK signals (normalized to GAPDH), obtained from three independent CTB preparations.

Supplementary Figure 3. Full-length Western blots are shown. Boxed areas depict signals shown in the regular figures.

Supplement to Figure 1d upper panel:

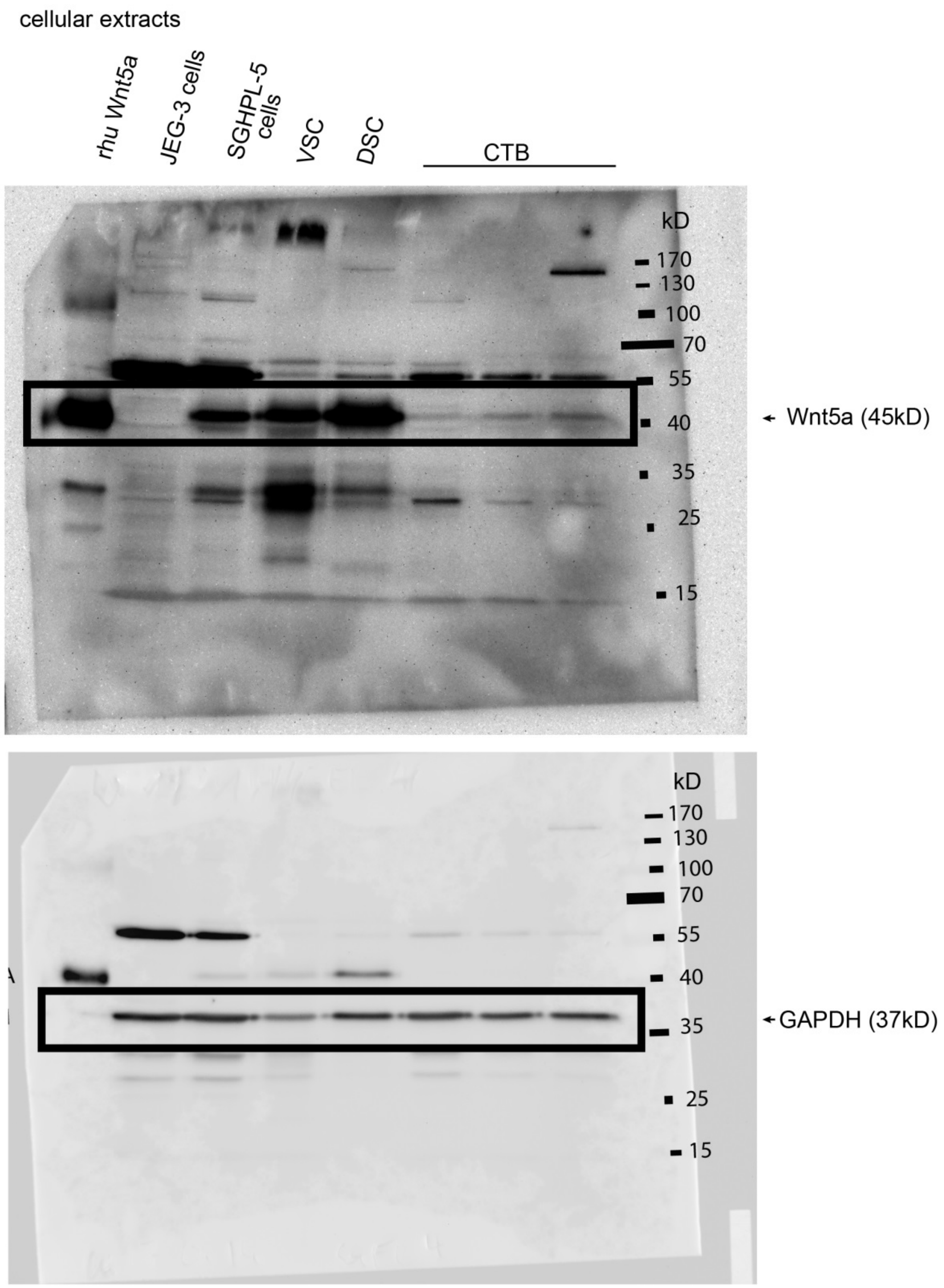

Supplement to Figure 1d lower panel, left picture:

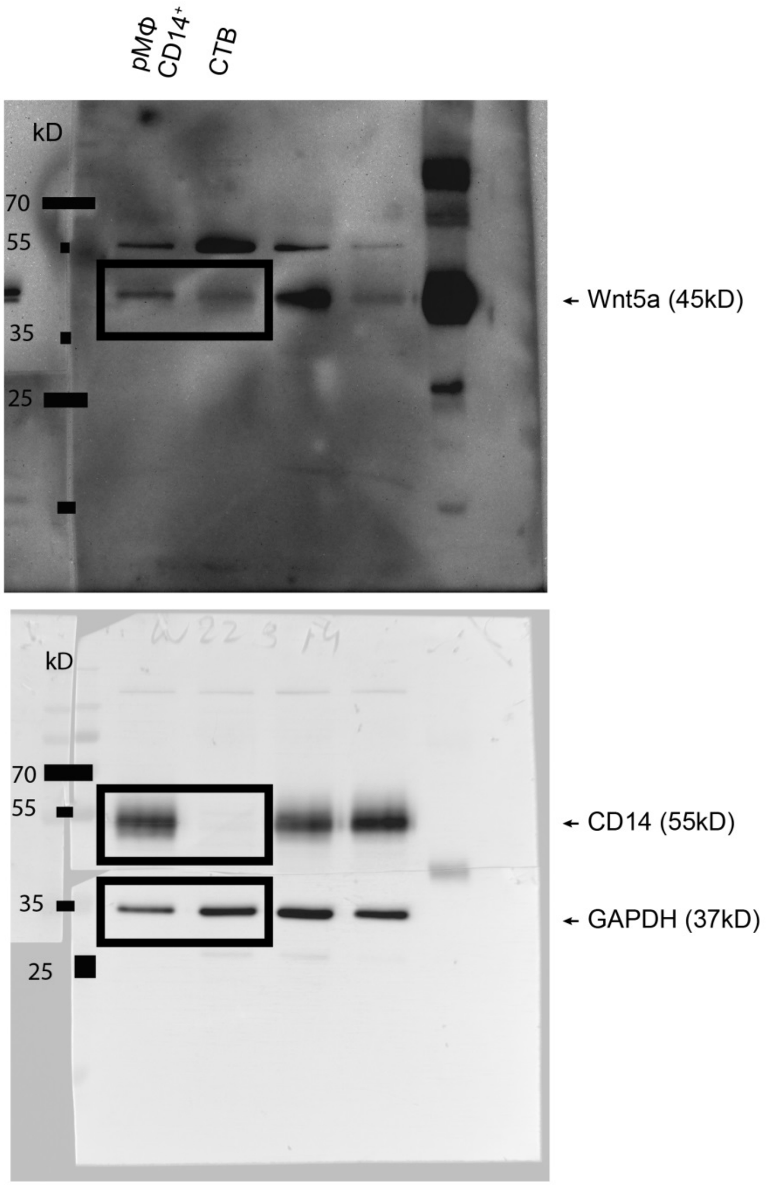

Supplement to Figure 1d lower panel, right picture:

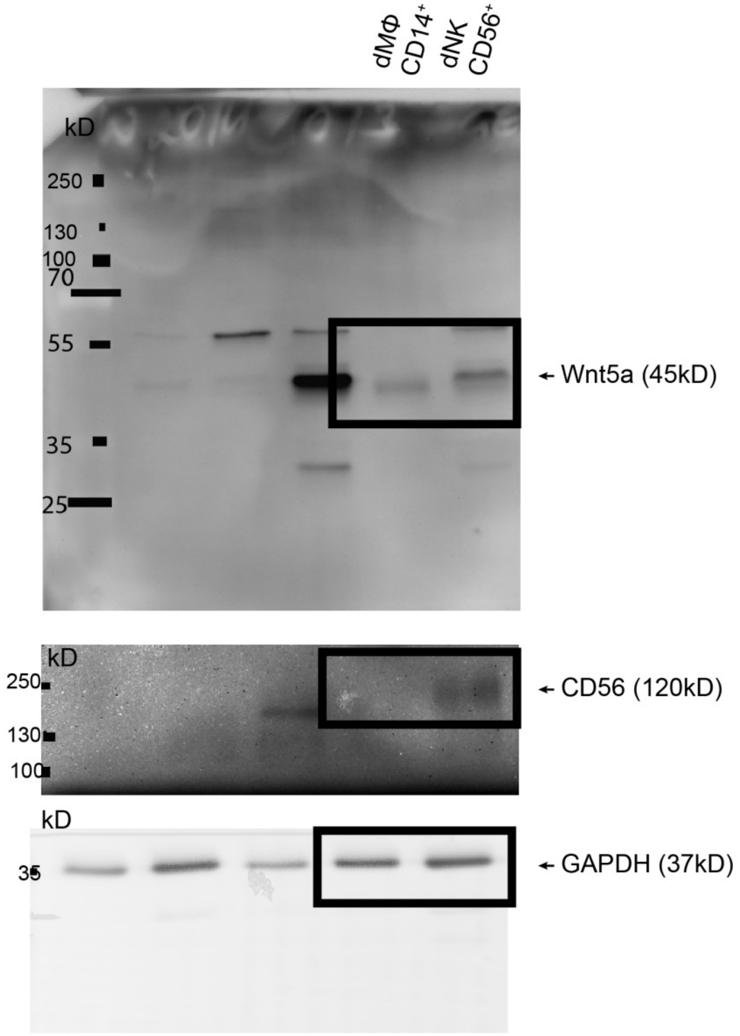

Supplement to Figure 1e:

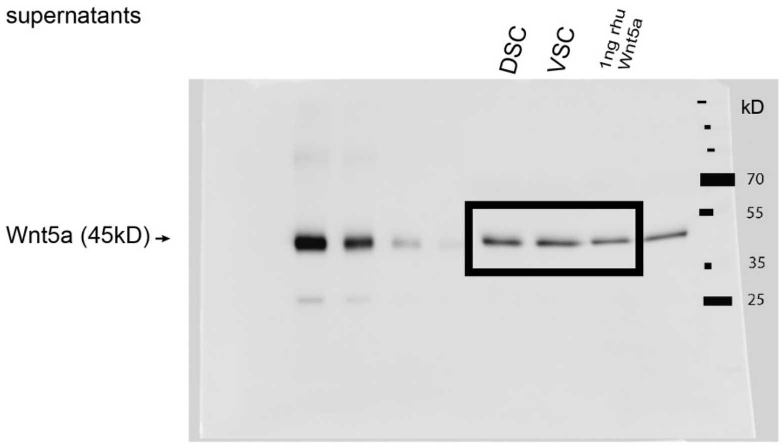

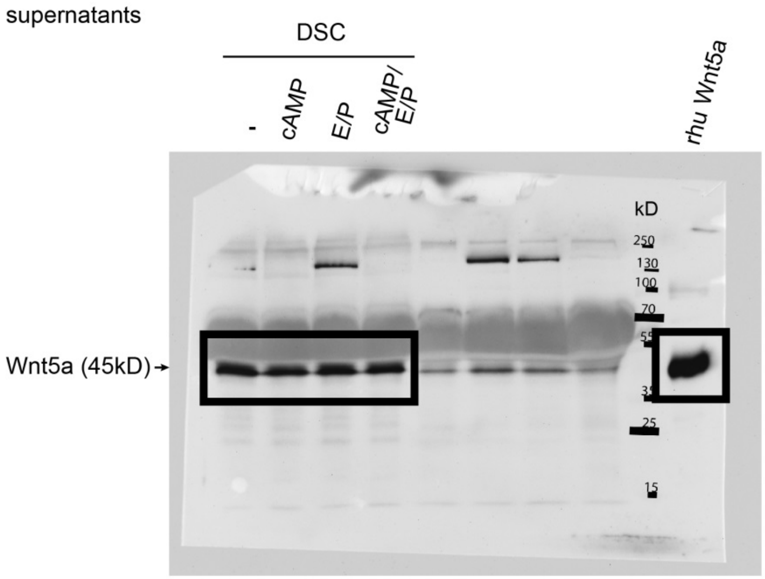

Supplement to Figure 2b:

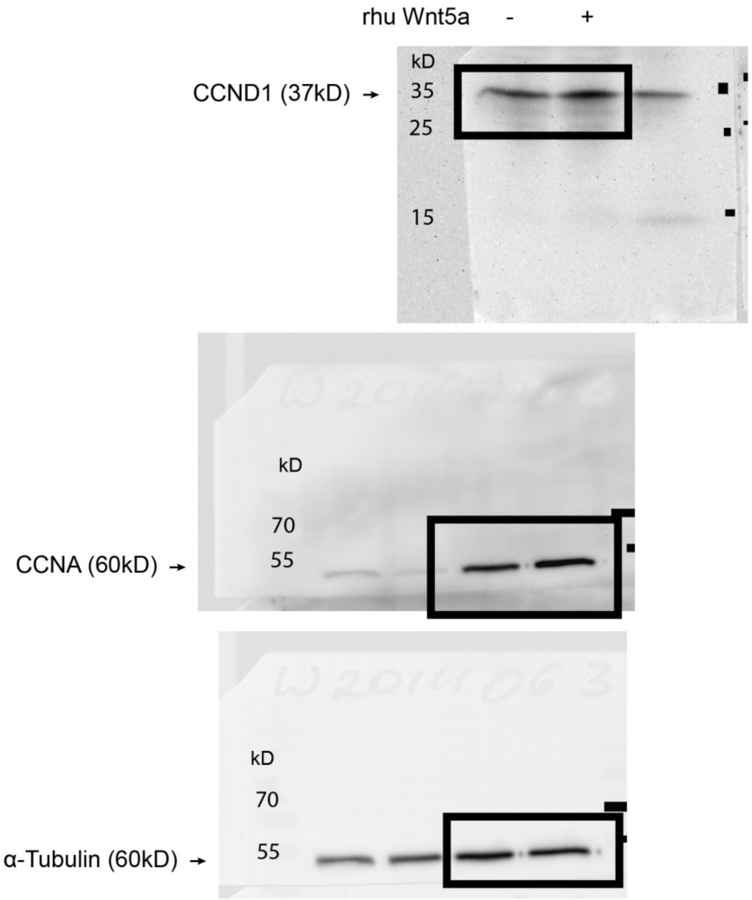

Supplement to Figure 2f:

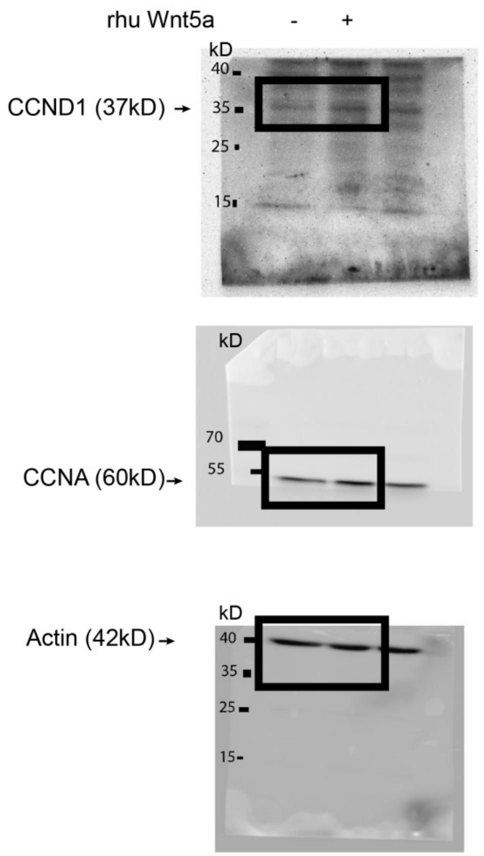

Supplement to Figure 3a:

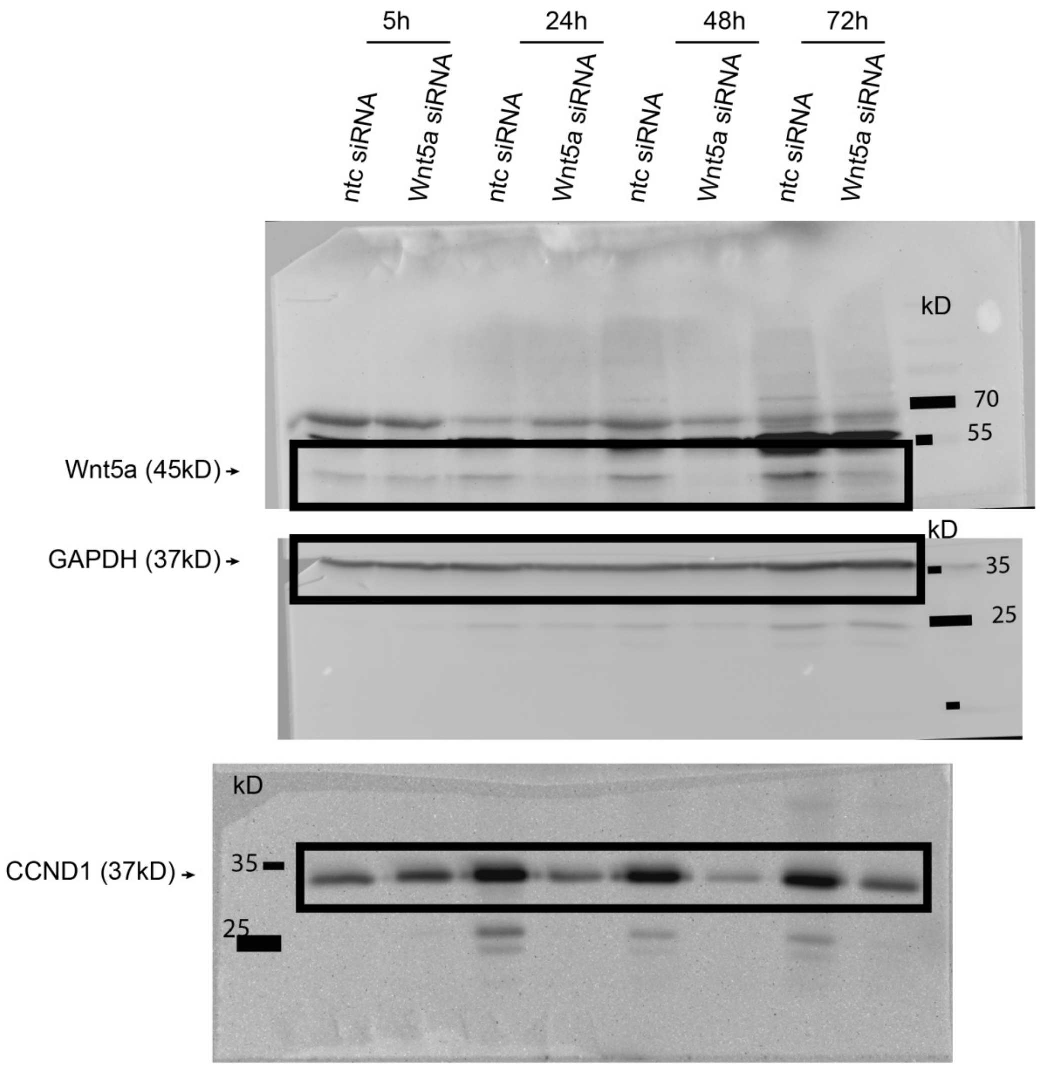

Supplement to Figure 3c:

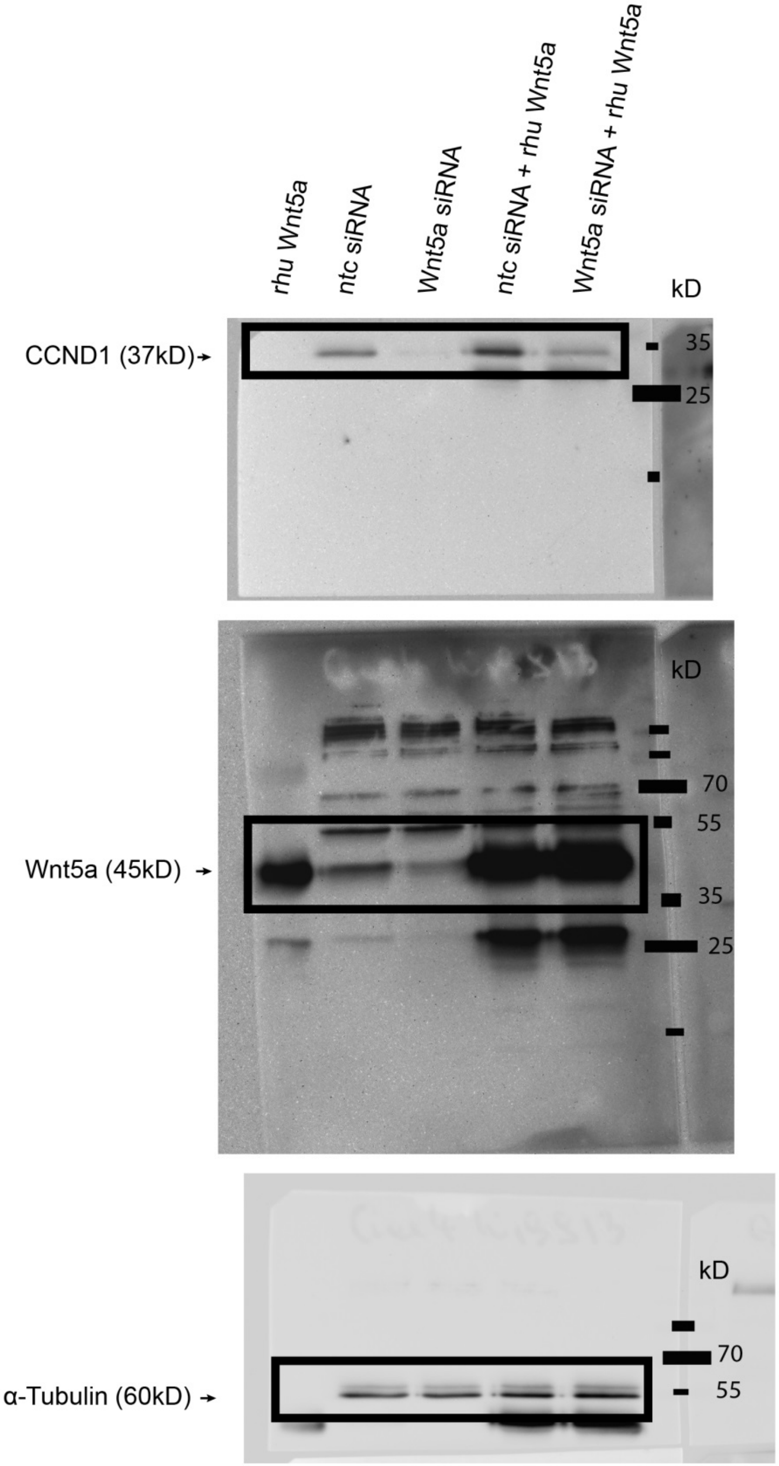

Supplement to Figure 4a:

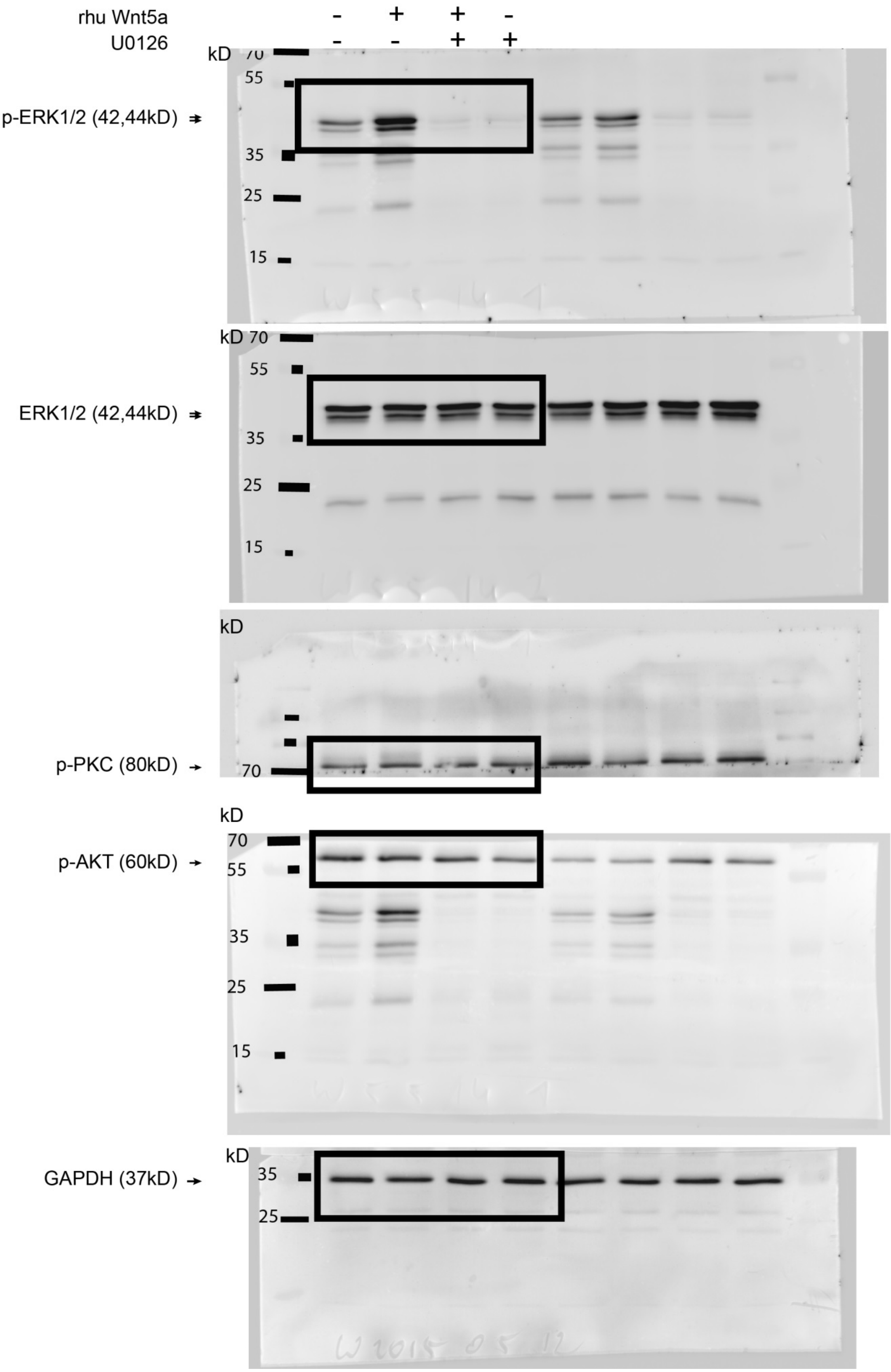

Supplement to Figure 4c:

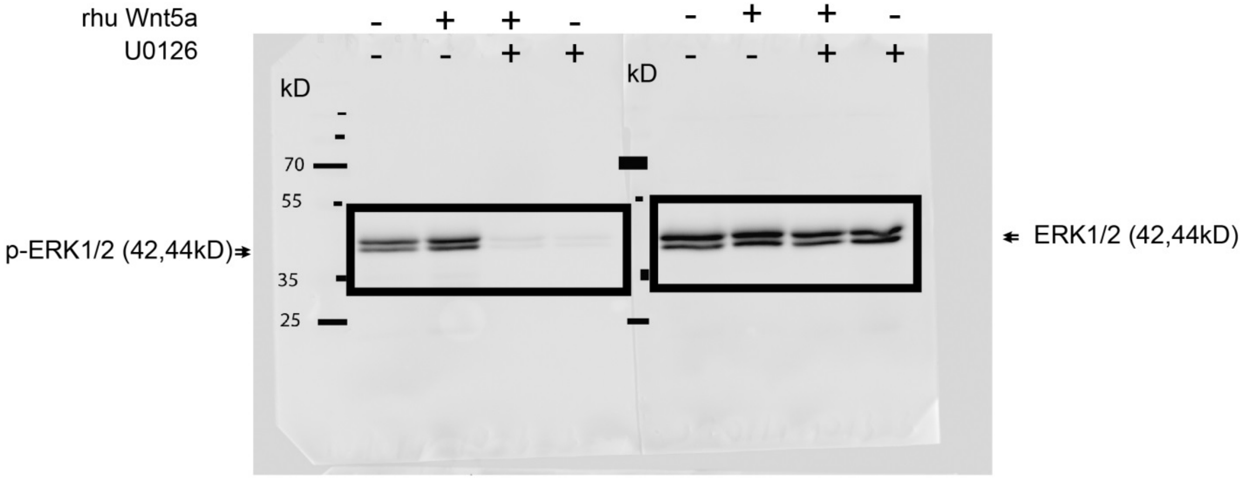

Supplement to Figure 5b:

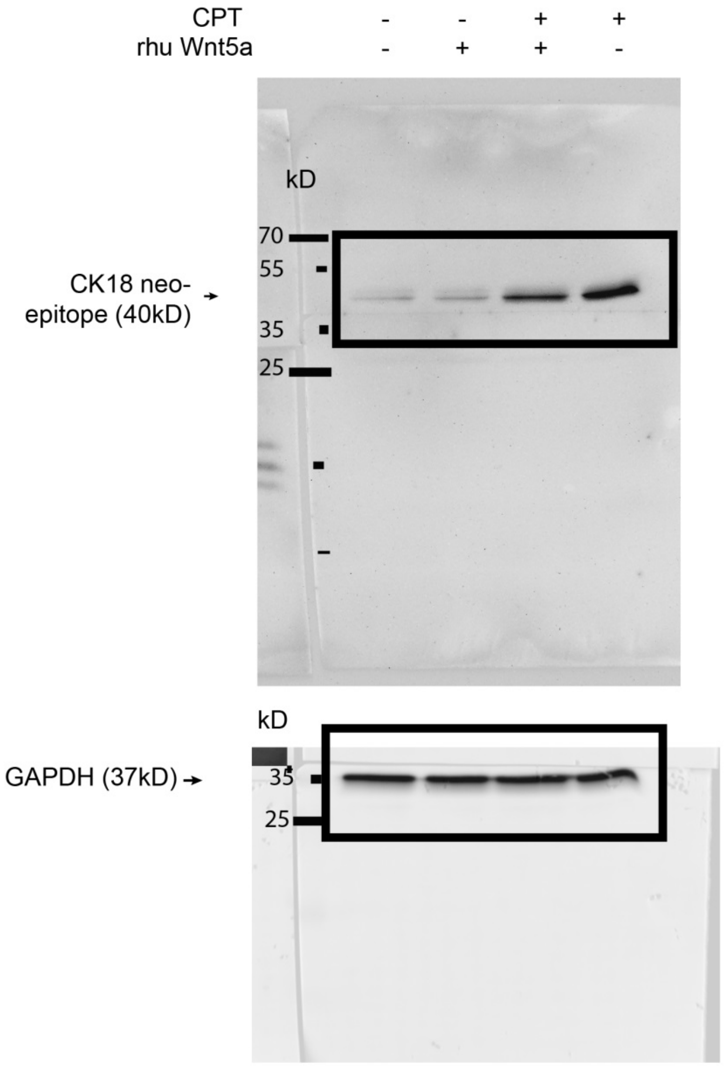

Supplement to Figure 5c:

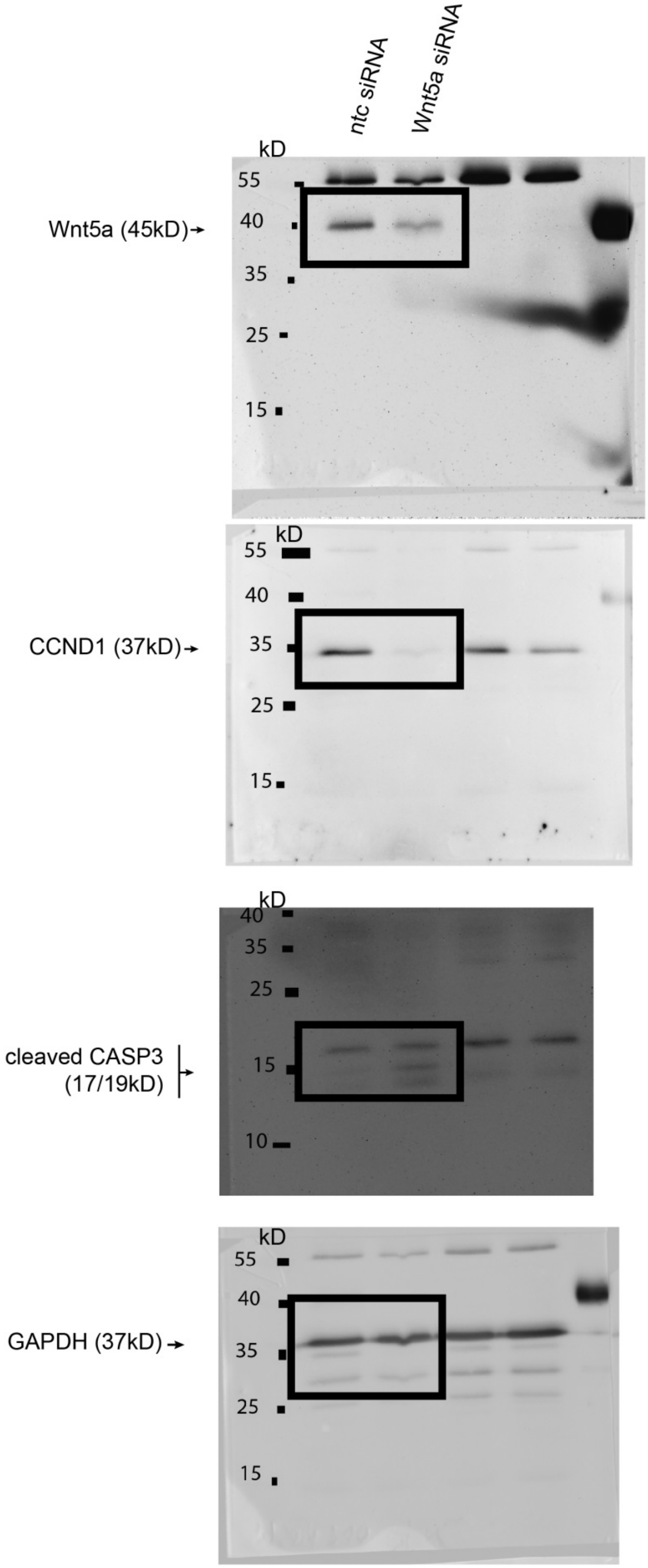

Supplement to Figure 6a:

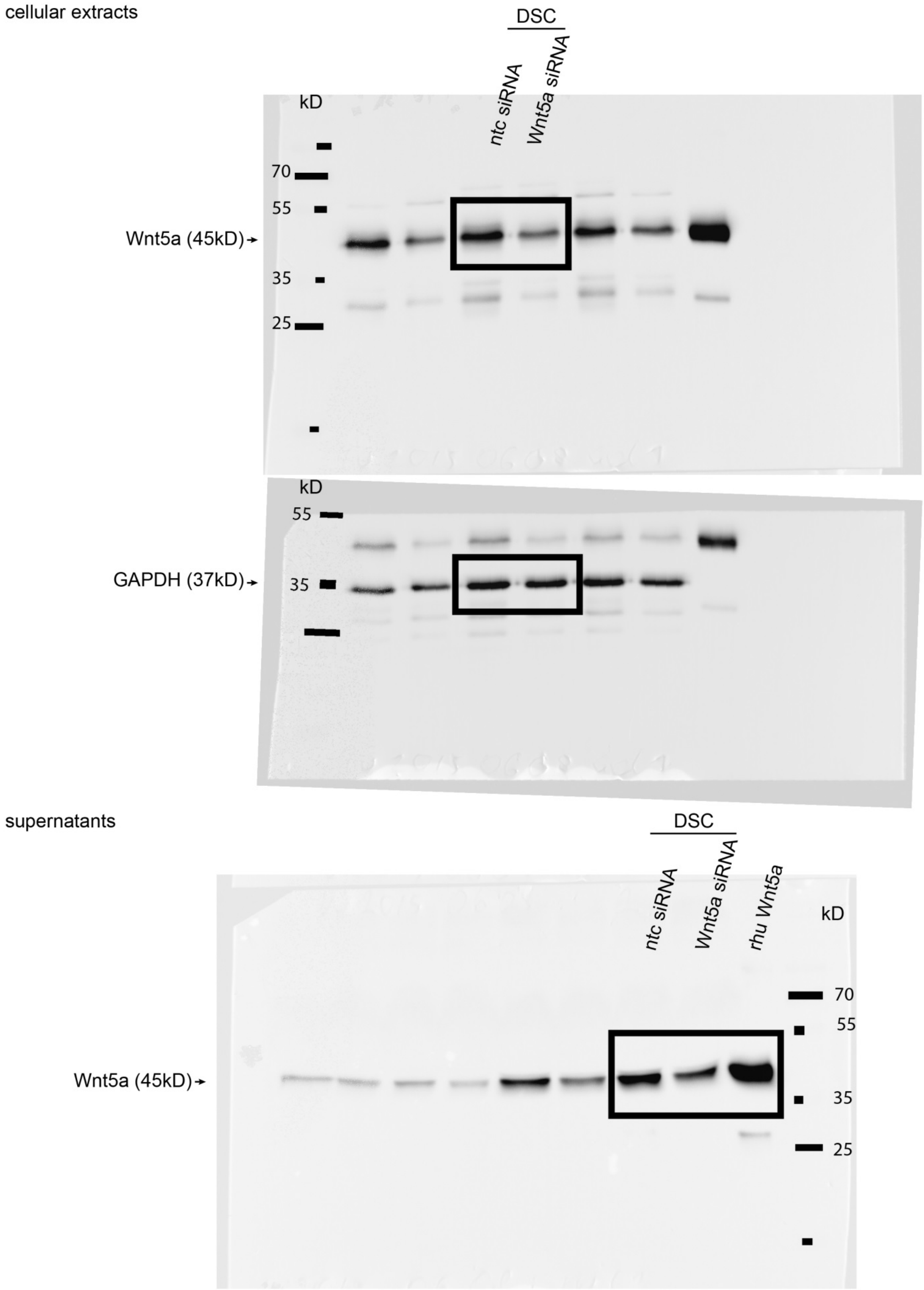

Supplement to Figure 6a:

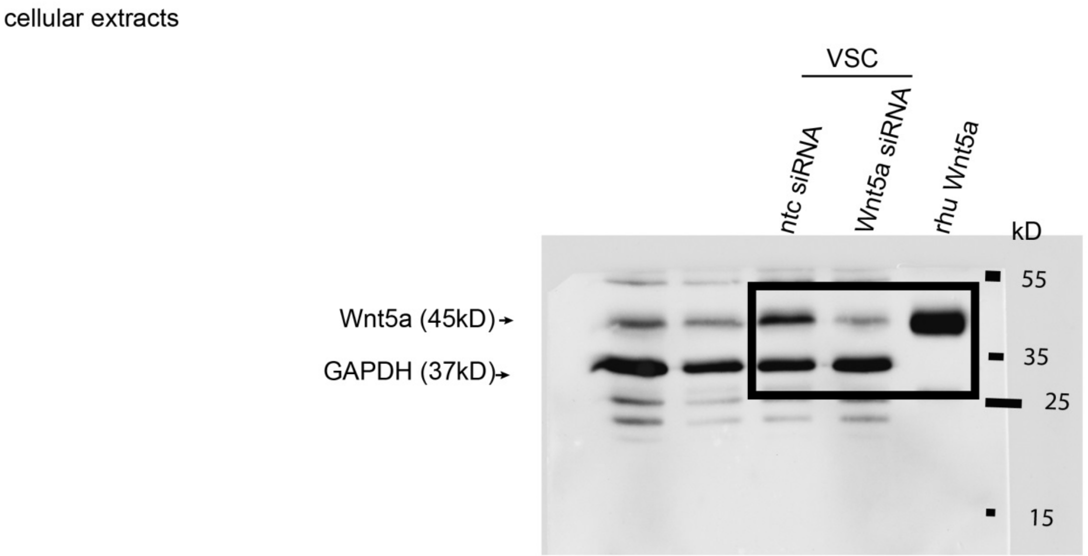

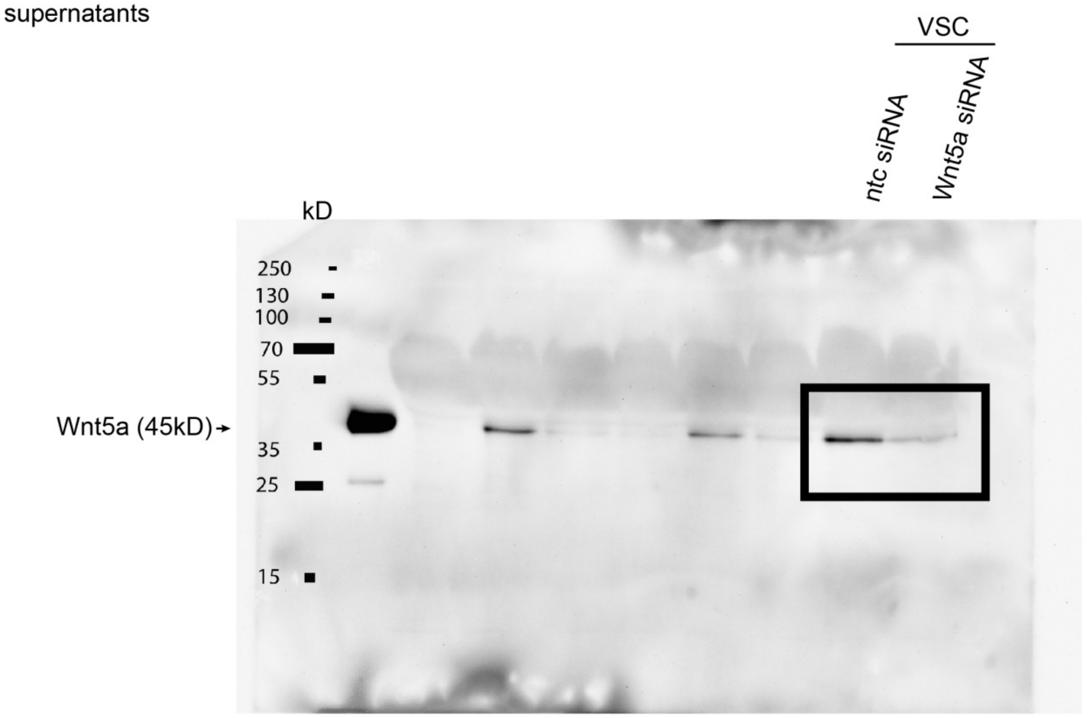

Supplement to Figure 6b:

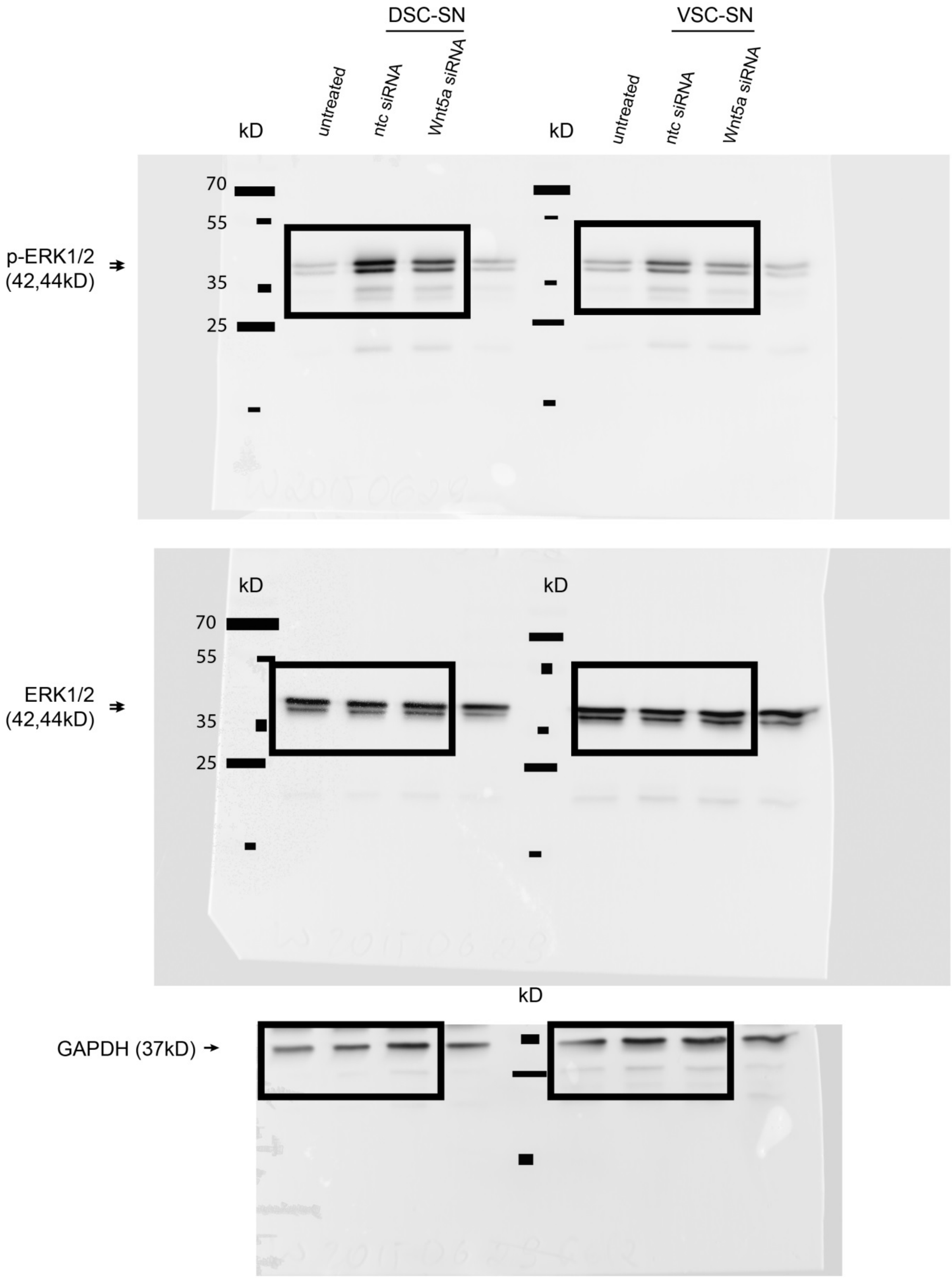

Supplement: Supplementary Information [file srep28127-s1.pdf]
